# Modeling physics data with the generalized Marshall-Olkin Kumaraswamy distribution

**DOI:** 10.1371/journal.pone.0329568

**Published:** 2026-02-17

**Authors:** Selim Gündüz, Egemen Ozkan, Kadir Karakaya

**Affiliations:** 1 Department of Business Administration, Faculty of Business, Adana Alparslan Türkeş Science and Technology University, Adana, Türkiye; 2 Department of Statistics, Yildiz Technical University, İstanbul, Türkiye; 3 Department of Statistics, Selcuk University, Konya, Türkiye; University of the Punjab, PAKISTAN

## Abstract

In this paper, a new distribution defined on a bounded interval is introduced, and its main properties, such as moments, Lorenz, and Bonferroni curves, are examined. The unknown parameters of the proposed distribution are estimated using several techniques, including maximum likelihood, least squares, weighted least squares, Anderson–Darling, Cramér–von Mises, maximum product spacing, right-tail Anderson–Darling, minimum spacing absolute distance, and minimum spacing absolute-log distance methods. The performance of these estimation methods is evaluated through Monte Carlo simulations under different parameter scenarios. Additionally, a new quantile regression model based on the proposed distribution is developed, offering greater flexibility for modeling bounded dependent variables. The capability of the proposed distribution to represent various hazard rate shapes, such as inverted-bathtub, bathtub, increasing, decreasing, constant, and increasing–decreasing–increasing, is to demonstrate its applicability and flexibility in real data analyses, particularly in cases where traditional models may underperform. Four different real-data applications from the fields of medicine, politics, physics, and education are presented to demonstrate that the proposed model is used as a strong alternative to the well-known Beta and Kumaraswamy distributions in modeling bounded data. The study provides a robust statistical tool for the analysis of bounded data, with potential applications in datasets related to medicine, politics, physics, and educational sciences.

## 1 Introduction

Statistical data analysis is fundamental to empirical research and applied sciences, driving progress across a wide range of fields such as econometrics, biomedical engineering, and environmental sustainability. The increasing complexity and size of datasets in these fields have driven the development of statistical methods for extracting reliable and interpretable insights. The lifetime statistical distributions such as exponentiated exponential [1], cubic rank transmuted generalized Gompertz [2], transmuted lower record type power function [3], Gumbel Marshall-Olkin Lomax [4], extended odd Weibull exponential [5], Odd-Lindley half logistic [6], Marshall-Olkin kappa [7], Kumaraswamy generalized kappa [8], and exponentiated additive Weibull [9] provided flexibility in modeling lifetime data. Despite these methodological advances, a significant gap persists in the statistical literature: there is a lack of distributions specifically designed for bounded data, which are commonly encountered in domains such as clinical trial results, financial ratios, mortality ratios, and ecological measurements. Although the beta and Kumaraswamy [10] distributions are widely used for modeling bounded data, they may not always be flexible enough to model complex data structures. In this context, new unit distribution models are derived from existing lifetime distributions through various transformation methods. Some of the recently proposed unit interval distributions based on existing distributions are as follows. The unit Weibull (UW) [11], unit Burr XII (UBXII) [12], unit log-log [13], unit Birnbaum-Saunders [14], unit inverse Gaussian [15], log-weighted exponential [16], and unit Chen [17] distributions are derived by using the transformation exp(−X), the unit Lindley (UL) [18] distribution is obtained by the transformation Y=X/(X+1). The unit improved second-degree Lindley [19], logit Slash [20], arcsecant hyperbolic normal [21], and transmuted unit Rayleigh (UR) [22] distributions are among the unit distributions obtained through other transformation methods.

In addition to generating new unit distributions, another important aspect of bounded data modeling is regression analysis. Regression analysis is a fundamental method that examines the relationship between the dependent variable and the independent variables, allowing for inference and predictive modeling beyond descriptive statistics. However, traditional regression models often struggle with data confined within specific intervals because standard distributions such as Gaussian do not adequately handle boundary constraints. This limitation highlights the essential requirement for new bounded probability distributions and corresponding regression frameworks. Accordingly, new regression approaches have been developed to model bounded data. These approaches provide a more flexible modeling framework compared to classical regression methods by considering the fact that the dependent variable is within the (0,1) interval. In this regard, the beta [23], Kumaraswamy (KW) [24], UBXII [12], log-extended exponential geometric [25], unit Burr-Hatke [26], and unit log-log [13] regression models are introduced as alternatives to classical regression models.

This study aims to propose a new and flexible distribution as an alternative to the well-known bounded models, such as the KW, beta, UL, UBXII, UW, and UR distributions. Although the existing unit distributions in the literature are used to model various types of bounded data, they are generally limited to representing only particular hazard rate shapes. Moreover, the proposed distribution provides substantial flexibility in modeling various datasets by representing diverse hazard rate shapes, including inverse-bathtub, bathtub, increasing, decreasing, constant, and increasing–decreasing–increasing. This regression model provides flexible modeling of dependent variables defined on the bounded data and extends data analysis by incorporating covariate effects. Finally, the efficiency of the proposed new distribution, compared to existing bounded distributions, is demonstrated through real data applications from the fields of political science, physics, medicine, and educational sciences. The findings confirm that the developed model provides a robust and innovative alternative to the existing bounded distributions and regression models in the literature.

The rest of the paper is structured as follows: In Sect 2, the generalized Marshall-Olkin Kumaraswamy (GMOKW) distribution is introduced, and its distributional properties, including moments, Lorenz, and Bonferroni curves, are investigated. Sect 3 provides an overview of nine different estimation methods. In Sect 4, a Monte Carlo simulation study is conducted to evaluate the performance of the estimators. In Sect 5, the new regression model is defined, and the unknown regression parameters are estimated via maximum likelihood methodology. In Sect 6, real data applications are presented to compare the proposed distribution and regression model with existing models by using selection criteria. In the final section, the study is concluded and key findings are summarized.

## 2 The generalized Marshall-Olkin Kumaraswamy distribution

In this section, the new bounded distribution is introduced. Firstly, the generalized Marshall-Olkin family introduced by [27] is described.The cumulative distribution function (cdf) and the probability density function (pdf) of the generalized Marshall-Olkin distribution family are given respectively, as

$$F\left(y\right)=\frac{\lambda G\left(y\right)+\left(1-\lambda \right){\left[G\left(y\right)\right]}^{2}}{\alpha +\left(1-\alpha \right)G\left(y\right)}$$ (1)

and

$$f\left(y\right)=\frac{\left(1-\alpha \right)\left(1-\lambda \right){\left[G\left(y\right)\right]}^{2}+2\alpha \left(1-\lambda \right)G\left(y\right)+\alpha \lambda }{{\left[\alpha +\left(1-\alpha \right)G\left(y\right)\right]}^{2}}g\left(y\right)$$ (2)

where α∈(0,1)-2pt,λ∈(0,1) and g(y) are shape parameters and G(y) are demonstrated the pdf and cdf functions of the baseline distribution, respectively. Assume that the random variable *Y* follows the KW distribution proposed by [10], with its cdf and pdf are given by,

$$G\left(y;\kappa ,\gamma \right)=1-{\left(1-y^{\kappa }\right)}^{\gamma }$$ (3)

and

$$g\left(y;\kappa ,\gamma \right)=\gamma \kappa y^{\kappa -1}{\left(1-y^{\kappa }\right)}^{\gamma -1}$$ (4)

where κ,γ>0 are shape parameters and 0 < *y* < 1. By substituting g(y;κ,γ) and G(y;κ,γ) into Eqs (1) and (2), we obtain the GMOKW. The cdf and pdf functions for the new distribution can be written as follows:

$$F\left(y;\alpha ,\lambda ,\kappa ,\gamma \right)=\frac{\lambda \left(1-{\left(1-y^{\kappa }\right)}^{\gamma }\right)+\left(1-\lambda \right){\left(1-{\left(1-y^{\kappa }\right)}^{\gamma }\right)}^{2}}{\alpha +\left(1-\alpha \right)\left(1-{\left(1-y^{\kappa }\right)}^{\gamma }\right)}$$ (5)

and

$$f\left(y;\alpha ,\lambda ,\kappa ,\gamma \right)=\left[\frac{\left(1-\alpha \right)\left(1-\lambda \right){\left(1-{\left(1-y^{\kappa }\right)}^{\gamma }\right)}^{2}+2\alpha \left(1-\lambda \right)\left(1-{\left(1-y^{\kappa }\right)}^{\gamma }\right)+\alpha \lambda }{{\left\{\alpha +\left(1-\alpha \right)\left(1-{\left(1-y^{\kappa }\right)}^{\gamma }\right)\right\}}^{2}}\right]\times \kappa \gamma y^{\kappa -1}{\left(1-y^{\kappa }\right)}^{\gamma -1}$$ (6)

where α,λ∈(0,1),κ,γ>0 and 0 < *y* < 1. In Fig 1, the pdf plots for different combinations of parameter values of the GMOKW are presented. Some shapes show a unimodal structure, initially increasing and then decreasing, while others demonstrate a monotonically decreasing or J-shaped increasing structure. In this case, the pdf plots shows that the GMOKW distribution has flexible shapes for different combinations of parameters.

**Fig 1:**
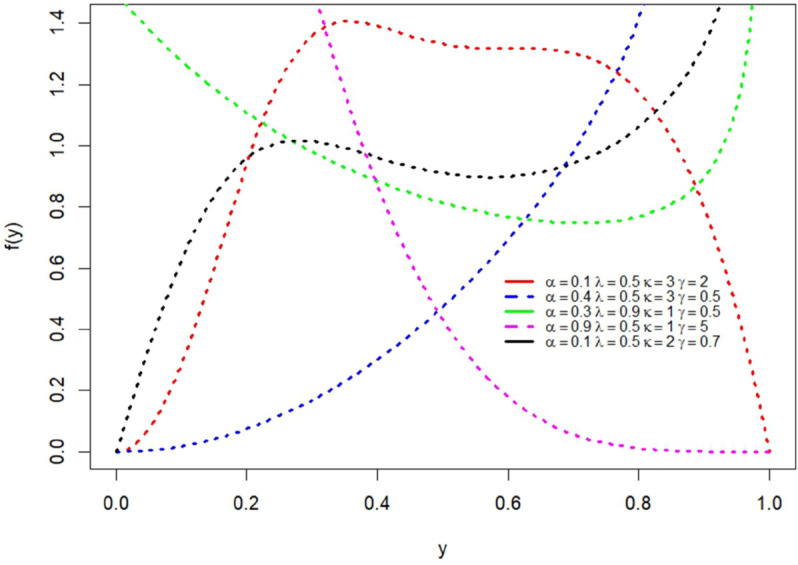
The pdf plots for different parameter values.

The hazard rate function (hrf) for the new distribution obtained is written:


$$h\left(y;\alpha ,\lambda ,\kappa ,\gamma \right)=\frac{\left(1-\alpha \right)\left(1-\lambda \right){\left[1-{\left(1-y^{\kappa }\right)}^{\gamma }\right]}^{2}+2\alpha \left(1-\lambda \right)\left[1-{\left(1-y^{\kappa }\right)}^{\gamma }\right]+\alpha \lambda }{\left\{\alpha +\left(1-\lambda \right)\left[1-{\left(1-y^{\kappa }\right)}^{\gamma }\right]\right\}\left\{\alpha +\left(1-\alpha \right)\left[1-{\left(1-y^{\kappa }\right)}^{\gamma }\right]\right\}}\;\;\times \frac{\kappa \gamma y^{\kappa -1}{\left(1-y^{\kappa }\right)}^{\gamma -1}}{{\left(1-y^{\kappa }\right)}^{\gamma }}.$$

**Fig 2:**
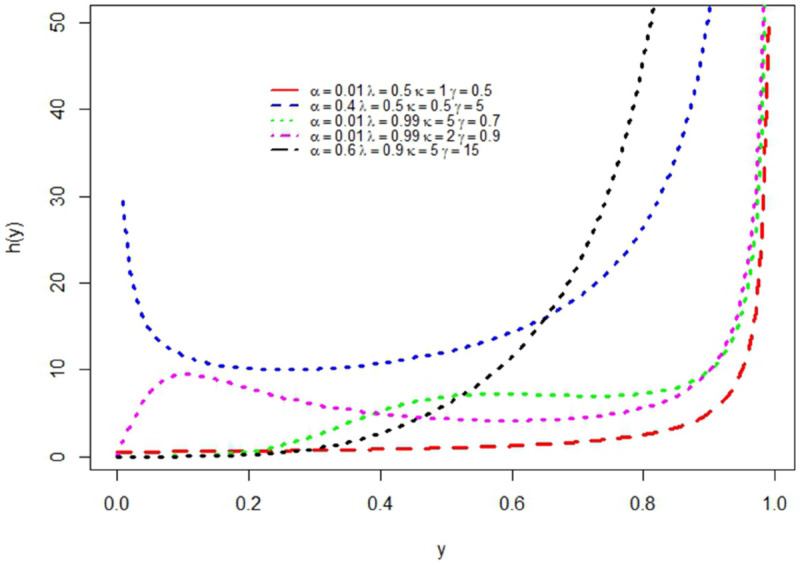
The hrf plots for different parameter values.

Fig 2 provides hrf plots for different combinations of parameters. Since the GMOKW distribution has hrfs with the shape of an upside-down bathtub, bathtub-shaped, increasing, decreasing, constant, and increasing-decreasing-increasing as depicted in Fig 2 for different parameter values, it can be regarded as a flexible distribution for modeling.

### 2.1 Series expansion and analytical representation for GMOKW distribution

For any *y* and *α* satisfying (1−α)[1−G(y)]∈(0,1), the pdf of the GMOKW can be expressed as following series expansion:


$$f\left(y\right)=g_{0}\left(y\right)+\sum_{i=0}^{+\infty }\sum_{m=0}^{k+1}u_{k,m}g_{m}\left(y\right)$$

where $g_{m}\left(y\right)=\left(m+1\right)g\left(y\right){\left[G\left(y\right)\right]}^{m}$, $u_{k,m}=\left(\lambda -\alpha \right)\left(\begin{array}{c} k+1\\ m \end{array}\right){\left(1-\alpha \right)}^{k}{\left(-1\right)}^{m}$, g(y) and G(y) are pdf and cdf of KW distribution, as given in Eqs (3) and (4).

### 2.2 Moments

If the random variable *Y* has a GMOKW distribution, the *rth* moment is obtained by,


$$E\left[Y^{r}\right]={Y_{0}}^{r}+\sum_{k=0}^{+\infty }\sum_{m=0}^{k+1}u_{k,m}Y_{m}^{r}$$

where $Y_{0}^{r}=\kappa \gamma \int_{0}^{1}y^{r+\kappa -1}(1-y^{\kappa }{)}^{\gamma }dy\;\text{and}\;Y_{m}^{r}=(m+1)\kappa \gamma \int_{0}^{1}y^{r+\kappa -1}(1-y^{\kappa }{)}^{\gamma -1}{\left[1-(1-y^{\kappa }{)}^{\gamma }\right]}^{m}dy.$ Then *rth* moment is easily computed by


E[Yr]=Γ(rκ+1)Γ(γ+1)Γ(rκ+γ+1)+∑k=0∞∑m=0k+1∑i=0m(λ−α)(1−α)κ(k+1m)(mi)×(m+1)(−1)m+iγΓ(γ(i+1))Γ(rκ+1)Γ(rκ+κ+κi+1),

where $\Gamma \left(\delta \right)=\int_{0}^{\infty }x^{\delta -1}e^{-x}dx$ is defined as gamma function. The skewness (S), and kurtosis (K) coefficients for the GMOKW distribution are obtained by using the first four moments as follows:


$$S=\frac{\Upsilon_{1}-3\Upsilon_{1}\Upsilon_{2}+2\Upsilon_{1}^{3}}{{\left(\Upsilon_{2}-\Upsilon_{1}^{2}\right)}^{\frac{3}{2}}},$$

$$K=\frac{\Upsilon_{4}-4\Upsilon_{1}\Upsilon_{3}+6\Upsilon_{2}\Upsilon_{1}^{2}-3\Upsilon_{1}^{4}}{{\left(\Upsilon_{2}-\Upsilon_{1}^{2}\right)}^{2}}$$

where


Υj=Γ(jκ+1)Γ(γ+1)Γ(jκ+γ+1)+∑k=0∞∑m=0k+1∑i=0m(λ−α)(1−α)κ(κ+1m)(mi)×(m+1)(−1)m+iγΓ(λ(i+1))Γ(jκ+1)Γ(jκ+κ+κi+1),j=1,2,3,4

Table 1 provides the variance, kurtosis, and skewness coefficients for various α,λ,κ and *γ* values. According to Table 1, it is clearly seen that as the α,λ and *γ* values increase, the expected value also increases. For small parameter values, the distribution exhibits right skewness and leptokurtosis, whereas larger *γ* values result in a transition towards left skewness and platykurtosis. For larger κ values, the skewness and kurtosis of the distribution increases. These results demonstrate that the GMOKW distribution can model different data structures through its shape parameters. This indicates that the distribution has the capability to be applied to a wide range of real-world datasets. Additionally, the pdf plots in Fig 1 are consistent with these results, and the graphical evidence supports the ability of the distribution to model different types of datasets.

**Table 1:** Expected value, variance. S and K for different parameter.

α	λ	κ	γ	E(X)	V	S	K
0.1	0.2	0.5	0.75	0.3804	0.1094	5.5444	26.4592
0.5	0.2	0.5	0.75	0.4891	0.1026	6.7415	29.0992
0.9	0.2	0.5	0.75	0.5527	0.0971	7.3589	32.7180
0.2	0.1	0.5	0.75	0.4469	0.1045	6.4446	27.4727
0.2	0.5	0.5	0.75	0.3217	0.1039	5.6175	26.0822
0.2	0.9	0.5	0.75	0.1964	0.0720	7.5858	28.0136
0.5	0.7	2	5	0.3426	0.0302	51.7709	17.7645
0.7	0.5	2	5	0.3927	0.0296	58.2789	19.1531
0.5	0.7	10	2	0.8515	0.0089	249.6621	121.0936
0.5	0.9	1	0.4	0.6194	0.0990	6.3548	39.3378
0.5	0.9	1	15	0.0475	0.0027	337.1826	15.5915

The *rth* incomplete moment of the GMOKW distribution is given by


$$E\left[Y^{r}\right]=\int_{0}^{x}y^{r}g\left(y\right)dy\;=\gamma \beta_{x}\left(\frac{r}{k}+1,\gamma \right)+\gamma \sum_{k=0}^{\infty }\sum_{m=0}^{k+1}\sum_{i=0}^{m}\left(\lambda -\alpha \right){\left(1-\alpha \right)}^{\kappa }\left(\begin{array}{c} \kappa +1\\ m \end{array}\right)\left(\begin{array}{c} m\\ i \end{array}\right)\;\;\times \left(m+1\right){\left(-1\right)}^{m+i}\beta_{x}\left(\lambda \left(i+1\right),\frac{r}{\kappa }+1\right),$$

where $\beta_{x}\left(a,b\right)$ is an incomplete beta function defined as $\beta_{x}\left(a,b\right)=\int_{0}^{x}t^{a-1}{\left(1-t\right)}^{b-1}dt$. The detailed information about the incomplete beta function can be found in [28].

### 2.3 Lorenz and Bonferroni curve

The Lorenz and Bonferroni curves are used to analyze data in economics, medical sciences, business and reliability. Accordingly, Lorenz and Bonferroni curves for the GMOKW distribution are given by respectively,


L(p)=1μ∫0qyg(y)dy=γβq(1κ+1,γ)+γ∑k=0∞∑m=0k+1∑i=0m(λ−α)(1−α)κ(κ+1m)(mi)×(m+1)(−1)m+iβq(λ(i+1),1κ+1)

and


B(p)=1μp∫0qyg(y)dy=γμpβq(1κ+1,γ)+γ∑k=0∞∑m=0k+1∑i=0m(λ−α)(1−α)κ(κ+1m)(mi)×(m+1)(−1)m+iβq(λ(i+1),1κ+1)

where *p* indicates the quantil function and *μ* is the first moment.

### 2.4 Quantile function

The quantile function for the GMOKW distribution can be obtained by

$$Q(p;\alpha ,\lambda ,\kappa ,\gamma )=-{\left(\frac{1}{2(-1+\lambda )}\left(-2+\lambda +p-p\alpha +\sqrt{4p\alpha +\lambda^{2}-2\lambda p-2\lambda p\alpha +p^{2}-2p^{2}\alpha +p^{2}\alpha^{2}}\right)\right)}^{\frac{1}{\beta }}+1$$ (7)

where p∈(0,1). This function is also used in simulation for generating data from the GMOKW distribution.

## 3 Parameter estimation

In this section, nine estimators are used to estimate the parameters of the GMOKW distribution, including maximum likelihood, least squares, weighted least squares, Anderson-Darling, Cramer-von Mises, maximum product spacing, right-tail Anderson-Darling, minimum spacing absolute distance, and minimum spacing absolute-log distance. Let $Y_{1},Y_{2},...,Y_{n}$ denote a sample taken from the GMOKW distribution and $Y_{\left(1\right)},Y_{\left(2\right)},...,Y_{\left(n\right)}$ represent the order statistics corresponding to this sample. Moreover, let *Y*_*i*_ denote the observerd values of the order statistics corresponding to $Y_{\left(i\right)}$ for i=1,2,...,n. Based on this information, the estimators for the GMOKW distribution are derived as follows.

### 3.1 Method of maximum likelihood

The likelihood function for the GMOKW distribution is given by

$$L\left(\alpha ,\lambda ,\kappa ,\gamma |𝐘\right)=\prod_{i=1}^{n}\left[\frac{\left(1-\alpha \right)\left(1-\lambda \right){\left(1-{\left(1-{y_{i}}^{\kappa }\right)}^{\gamma }\right)}^{2}+2\alpha \left(1-\lambda \right){\left(1-\left(1-{y_{i}}^{\kappa }\right)\right)}^{\gamma }+\alpha \lambda }{{\left\{\alpha +\left(1-\alpha \right){\left(1-\left(1-{y_{i}}^{\kappa }\right)\right)}^{\gamma }\right\}}^{2}}\right]\;\;\times \kappa \gamma {y_{i}}^{\kappa -1}{\left(1-{y_{i}}^{\kappa }\right)}^{\gamma -1}$$ (8)

By taking the logarithm of Eq (8), the log-likelihood function is obtained as follows.

$$\ell \left(\alpha ,\lambda ,\kappa ,\gamma |Y\right)=\sum_{i=1}^{n}\log\left(\left(1-\alpha \right)\left(1-\lambda \right){\left(1-{\left(1-{y_{i}}^{\kappa }\right)}^{\gamma }\right)}^{2}+2\alpha \left(1-\lambda \right){\left(1-\left(1-{y_{i}}^{\kappa }\right)\right)}^{\gamma }+\alpha \lambda \right)\;\;+\sum_{i=1}^{n}\log\left(\kappa \gamma {y_{i}}^{\kappa -1}{\left(1-{y_{i}}^{\kappa }\right)}^{\gamma -1}\right)-\sum_{i=1}^{n}\log\left(\alpha +\left(1-\alpha \right){\left(1-\left(1-{y_{i}}^{\kappa }\right)\right)}^{\gamma }\right)$$ (9)

The maximum likelihood estimators (MLEs) of the α,λ,κ,γ parameters are obtained by solving the following system of equations.


$$\frac{\partial \ell \left(\alpha ,\lambda ,\kappa ,\gamma |Y\right)}{\partial \alpha }=\sum_{i=1}^{n}\frac{2\left(1-\lambda \right)\left(1-{\left(1-{y_{i}}^{\kappa }\right)}^{\gamma }\right)+\lambda -\left(1-\lambda \right){\left(1-{\left(1-{y_{i}}^{\kappa }\right)}^{\gamma }\right)}^{2}}{\left(1-\alpha \right)\left(1-\lambda \right){\left(1-{\left(1-{y_{i}}^{\kappa }\right)}^{\gamma }\right)}^{2}+2\alpha \left(1-\lambda \right)\left(1-{\left(1-{y_{i}}^{\kappa }\right)}^{\gamma }\right)+\alpha \lambda }\;\;+\sum_{i=1}^{n}\frac{2{\left(1-y_{i}^{\kappa }\right)}^{\gamma }}{\alpha +\left(1-\alpha \right)\left(1-{\left(1-y_{i}^{\gamma }\right)}^{\gamma }\right)}=0$$

$$\frac{\partial \ell \left(\alpha ,\lambda ,\kappa ,\gamma |Y\right)}{\partial \lambda }=\sum_{i=1}^{n}\frac{\left(1-\alpha \right){\left(1-{\left(1-{y_{i}}^{\kappa }\right)}^{\gamma }\right)}^{2}+2\alpha \left(1-{\left(1-{y_{i}}^{\kappa }\right)}^{\gamma }\right)+\alpha }{\left(1-\alpha \right)\left(1-\lambda \right){\left(1-{\left(1-{y_{i}}^{\kappa }\right)}^{\gamma }\right)}^{2}+2\alpha \left(1-\lambda \right)\left(1-{\left(1-{y_{i}}^{\kappa }\right)}^{\gamma }\right)+\alpha \lambda }=0$$

$$\frac{\partial \ell \left(\alpha ,\lambda ,\kappa ,\gamma |Y\right)}{\partial \kappa }=\sum_{i=1}^{n}\frac{2\gamma \log\left(y_{i}\right){\left(1-{y_{i}}^{\kappa }\right)}^{\gamma }\left(1+\left(\alpha -1\right){\left(1-y_{i}^{\kappa }\right)}^{\gamma }\right)\left(\lambda -1\right)y_{i}^{\kappa }}{\left(y_{i}^{\kappa }-1\right)\left(\left(\lambda -1\right)\left(\alpha -1\right){\left(1-y_{i}^{\kappa }\right)}^{2\gamma }+\left(2\lambda -2\right){\left(1-y_{i}^{\kappa }\right)}^{\gamma }+\alpha -\lambda +1\right)}\;\;+\sum_{i=1}^{n}\frac{\kappa \log\left(y_{i}\right)y_{i}^{\kappa }\gamma -\kappa \log\left(y_{i}\right)+y_{i}^{\kappa }-1}{\left(y_{i}^{\kappa }-1\right)\kappa }+\sum_{i=1}^{n}\frac{2\left(\alpha -1\right){\left(1-y_{i}^{\kappa }\right)}^{\gamma }\gamma y_{i}^{\kappa }\log\left(y_{i}\right)}{\left(1+\left(\alpha -1\right){\left(1-y^{\kappa }\right)}^{\gamma }\right)\left(y_{i}^{\kappa }-1\right)}=0$$

$$\frac{\partial \ell \left(\alpha ,\lambda ,\kappa ,\gamma \right)}{\partial \gamma }=\sum_{i=1}^{n}\frac{2\log\left(1-{y_{i}}^{\kappa }\right)\left(1+\left(\alpha -1\right){\left(1-{y_{i}}^{\kappa }\right)}^{\gamma }\right)\left(\lambda -1\right){\left(1-{y_{i}}^{\kappa }\right)}^{\gamma }}{\left(\lambda -1\right)\left(\alpha -1\right){\left(1-{y_{i}}^{\kappa }\right)}^{2\gamma }+\left(2\lambda -2\right){\left(1-{y_{i}}^{\kappa }\right)}^{\gamma }+1+\alpha -\lambda }\;\;+\frac{1}{\gamma }\sum_{i=1}^{n}\left[1+\gamma \log\left(1-y_{i}^{\kappa }\right)\right]+2\sum_{i=1}^{n}\frac{2\left(\alpha -1\right){\left(1-y_{i}^{\kappa }\right)}^{\gamma }\log\left(1-y_{i}^{\kappa }\right)}{1+\left(\alpha -1\right){\left(1-y_{i}^{\kappa }\right)}^{\gamma }}=0.$$

### 3.2 Method of least squares

This method is utilized for parameter estimation in a mathematical model by reducing the sum of squared differences between the observed data and the predicted outcomes. The following equation can be minimized to find the least square estimator (LSE) of the parameter:

$$LSE\left(y_{i}\right)=\sum_{i=1}^{n}{\left[\frac{\lambda \left(1-{\left(1-{y_{\left(i\right)}}^{\kappa }\right)}^{\gamma }\right)+\left(1-\lambda \right){\left(1-{\left(1-{y_{\left(i\right)}}^{\kappa }\right)}^{\gamma }\right)}^{2}}{\alpha +\left(1-\alpha \right)\left(1-{\left(1-{y_{\left(i\right)}}^{\kappa }\right)}^{\gamma }\right)}-\frac{i}{n+1}\right]}^{2}.$$ (10)

The minimization of the function presented in Eq (10) is obtained by taking the partial derivatives with respect to each parameter and solving the resulting system of equations:


$$\frac{\partial LSE(y_{i})}{\partial \alpha }=\sum_{i=1}^{n}\frac{\left(\frac{\lambda z_{\left(i\right)}+(1-\lambda )z_{\left(i\right)}^{2}}{\alpha +(1-\alpha )z_{\left(i\right)}}-\frac{i}{n+1}\right)\left(\lambda z_{\left(i\right)}+(1-\lambda {)}^{2}z_{\left(i\right)}^{2}(1-z_{\left(i\right)})\right)}{{\left(\alpha +(1-\alpha )z_{\left(i\right)}\right)}^{2}}=0,$$

$$\frac{\partial LSE(y_{\left(i\right)})}{\partial \lambda }=\sum_{i=1}^{n}\frac{\left(\frac{\lambda z_{\left(i\right)}+(1-\lambda {)}^{2}z_{\left(i\right)}^{2}}{\alpha +(1-\alpha )z_{\left(i\right)}}-\frac{i}{n+1}\right)\left(z_{\left(i\right)}-2(1-\lambda )z_{\left(i\right)}^{2}\right)}{\alpha +(1-\alpha )z_{\left(i\right)}}=0,$$

$$\frac{\partial LSE(y_{i})}{\partial \kappa }=\sum_{i=1}^{n}[(\frac{\lambda (1-z_{\left(i\right)})\gamma y_{\left(i\right)}^{\kappa }\ln(y_{\left(i\right)})+2(1-\lambda {)}^{2}z_{\left(i\right)}(1-z_{\left(i\right)})\gamma y_{\left(i\right)}^{\kappa }\ln(y_{\left(i\right)})}{\left(1-y_{\left(i\right)}^{\kappa })(\alpha +(1-\alpha )z_{\left(i\right)}\right)}\;\;-\frac{\left(\lambda z_{\left(i\right)}+(1-\lambda {)}^{2}z_{\left(i\right)}^{2})(1-\alpha )(1-z_{\left(i\right)})\gamma y_{\left(i\right)}^{\kappa }\ln(y_{\left(i\right)}\right)}{(1-y_{\left(i\right)}^{\kappa })(\alpha +(1-\alpha )z_{\left(i\right)}{)}^{2}})\;\;\times (\frac{\lambda z_{\left(i\right)}+(1-\lambda {)}^{2}z_{\left(i\right)}^{2}}{\alpha +(1-\alpha )z_{\left(i\right)}}-\frac{i}{n+1})]=0.$$

$$\frac{\partial LSE(y_{i})}{\partial \gamma }=\sum_{i=1}^{n}[(\frac{-\lambda (1-z_{\left(i\right)})\ln(1-y_{\left(i\right)}^{\kappa })-2(1-\lambda {)}^{2}(1-z_{\left(i\right)})z_{\left(i\right)}\ln(1-y_{\left(i\right)}^{\kappa })}{\alpha +(1-\alpha )z_{\left(i\right)}}\;\;+\frac{\left(\lambda z_{\left(i\right)}+(1-\lambda {)}^{2}z_{\left(i\right)}^{2})(1-\alpha )(1-z_{\left(i\right)})\ln(1-y_{\left(i\right)}^{\kappa }\right)}{(\alpha +(1-\alpha )z_{\left(i\right)}{)}^{2}})\;\;\times (\frac{\lambda z_{\left(i\right)}+(1-\lambda {)}^{2}z_{\left(i\right)}^{2}}{\alpha +(1-\alpha )z_{\left(i\right)}}-\frac{i}{n+1})]=0.$$

where $z_{i}={\left(1-\left(1-y^{\kappa }\right)\right)}^{\gamma }$.

### 3.3 Methods of weighted least squares

The weighted least squares estimator (WLSE) are a minimization-based estimation method introduced as an alternative to LSE. The fundamental purpose of WLSE is to assign different weights to each observation, rather than giving equal weights to observations as in LSE, in order to estimate the parameters of a probability model. Then the WLSE of the parameter of the GMOKW distribution is obtained by minimizing the following equation.

$$WLSE\left(y_{i}\right)=\sum_{i=1}^{n}w_{i}{\left[\frac{\lambda \left(1-{\left(1-{y_{\left(i\right)}}^{\kappa }\right)}^{\gamma }\right)+\left(1-\lambda \right){\left(1-{\left(1-{y_{\left(i\right)}}^{\kappa }\right)}^{\gamma }\right)}^{2}}{\alpha +\left(1-\alpha \right)\left(1-{\left(1-{y_{\left(i\right)}}^{\kappa }\right)}^{\gamma }\right)}-\frac{i}{n+1}\right]}^{2}$$ (11)

where $w_{i}=\frac{{\left(n+1\right)}^{2}\left(n+2\right)}{i\left(n-i+1\right)}$. The function given in Eq (11) is minimized by solving the system of equations obtained by taking the partial derivatives with respect to the parameters and equating them to zero. The partial derivatives of the function with respect to the parameters are given by:


$$\frac{\partial LSE(y_{i})}{\partial \alpha }=\sum_{i=1}^{n}\left[w_{i}\frac{\left(\frac{\lambda z_{\left(i\right)}+(1-\lambda )z_{\left(i\right)}^{2}}{\alpha +(1-\alpha )z_{\left(i\right)}}-\frac{i}{n+1}\right)\left(\lambda z_{\left(i\right)}+(1-\lambda {)}^{2}z_{\left(i\right)}^{2}(1-z_{\left(i\right)})\right)}{{\left(\alpha +(1-\alpha )z_{\left(i\right)}\right)}^{2}}\right]=0,$$

$$\frac{\partial LSE(y_{i})}{\partial \lambda }=\sum_{i=1}^{n}\left[w_{i}\frac{\left(\frac{\lambda z_{\left(i\right)}+(1-\lambda {)}^{2}z_{\left(i\right)}^{2}}{\alpha +(1-\alpha )z_{\left(i\right)}}-\frac{i}{n+1}\right)\left(z_{\left(i\right)}-2(1-\lambda )z_{\left(i\right)}^{2}\right)}{\alpha +(1-\alpha )z_{\left(i\right)}}\right]=0,$$

$$\frac{\partial LSE(y_{i})}{\partial \kappa }=\sum_{i=1}^{n}[w_{i}(\frac{\lambda (1-z_{\left(i\right)})\gamma y_{\left(i\right)}^{\kappa }\ln(y_{\left(i\right)})+2(1-\lambda {)}^{2}z_{\left(i\right)}(1-z_{\left(i\right)})\gamma y_{\left(i\right)}^{\kappa }\ln(y_{\left(i\right)})}{\left(1-y_{\left(i\right)}^{\kappa })(\alpha +(1-\alpha )z_{\left(i\right)}\right)}\;\;-\frac{\left(\lambda z_{\left(i\right)}+(1-\lambda {)}^{2}z_{\left(i\right)}^{2})(1-\alpha )(1-z_{\left(i\right)})\gamma y_{\left(i\right)}^{\kappa }\ln(y_{\left(i\right)}\right)}{(1-y_{\left(i\right)}^{\kappa })(\alpha +(1-\alpha )z_{\left(i\right)}{)}^{2}})\;\;\times (\frac{\lambda z_{\left(i\right)}+(1-\lambda {)}^{2}z_{\left(i\right)}^{2}}{\alpha +(1-\alpha )z_{\left(i\right)}}-\frac{i}{n+1})]=0.$$

$$\frac{\partial LSE(y_{i})}{\partial \gamma }=\sum_{i=1}^{n}[w_{i}(\frac{-\lambda (1-z_{\left(i\right)})\ln(1-y_{\left(i\right)}^{\kappa })-2(1-\lambda {)}^{2}(1-z_{\left(i\right)})z_{\left(i\right)}\ln(1-y_{\left(i\right)}^{\kappa })}{\alpha +(1-\alpha )z_{\left(i\right)}}\;\;+\frac{\left(\lambda z_{\left(i\right)}+(1-\lambda {)}^{2}z_{\left(i\right)}^{2})(1-\alpha )(1-z_{\left(i\right)})\ln(1-y_{\left(i\right)}^{\kappa }\right)}{(\alpha +(1-\alpha )z_{\left(i\right)}{)}^{2}})\;\;\times (\frac{\lambda z_{\left(i\right)}+(1-\lambda {)}^{2}z_{\left(i\right)}^{2}}{\alpha +(1-\alpha )z_{\left(i\right)}}-\frac{i}{n+1})]=0,$$

where $z_{i}={\left(1-\left(1-y^{\kappa }\right)\right)}^{\gamma }$ is defined.

### 3.4 Method of Anderson-Darling

The Anderson-Darling estimator (ADE) is a method introduced to provide more accurate estimators in the presence of deviations from the assumed model. Accordingly, the ADE of the parameter of the GMOKW distribution is obtained by minimizing the following function.

$$ADE(y_{i})=-n-\frac{1}{n}\sum_{i=1}^{n}\left[\log\left(\frac{\lambda z_{i}+(1-\lambda )z_{i}^{2}}{\alpha +\left(1-\alpha \right)z_{i}}\right)+\log\left(1-\frac{\lambda z_{n-i-1}+(1-\lambda )z_{n-i-1}^{2}}{\alpha +\left(1-\alpha \right)z_{n-i-1}}\right)\right]$$ (12)

where $z_{i}={\left(1-\left(1-y^{\kappa }\right)\right)}^{\gamma }$ is defined. Therefore, the first derivatives of the function presented in Eq (12) are obtained with respect to the unknown parameters and equated to zero, resulting in the following system of nonlinear equations.


$$\frac{\partial ADE\left(y_{i}\right)}{\partial \alpha }=\sum_{i=1}^{n}\left[\left(2i-1\right)\left(\frac{1-z_{\left(i\right)}}{\alpha +\left(1-\alpha \right)z_{\left(i\right)}}+\frac{z\left(\lambda z_{\left(n-i-1\right)}-z_{\left(n-i-1\right)}-\lambda \right)}{\left(\lambda z-\alpha -z\right)\left((\alpha -1)z_{\left(n-i-1\right)}-\alpha \right)}\right)\right]=0,$$

$$\frac{\partial ADE\left(y_{i}\right)}{\partial \lambda }=\sum_{i=1}^{n}\left[\left(2i-1\right)\left(\frac{z_{\left(i\right)}}{(\lambda -1)z_{\left(i\right)}-\lambda }+\frac{z_{\left(i\right)}}{(\lambda -1)z_{\left(i\right)}-\alpha }\right)\right]=0,$$

$$\frac{\partial ADE(y_{i})}{\partial \kappa }=\sum_{i=1}^{n}[\frac{(2i-1)\log(y_{\left(i\right)})\;y_{\left(i\right)}^{\kappa }\gamma ((\lambda -1)(1-\alpha )(1-z_{\left(i\right)}{)}^{2}+(2\lambda -2)(1-z_{\left(i\right)})-\lambda +\alpha +1)z_{\left(i\right)}}{\left(1+(1-z_{\left(i\right)})z_{\left(i\right)})((1-\lambda )z_{\left(i\right)}-1)((1-z_{\left(i\right)})z_{\left(i\right)}\right)}]\;\;+\sum_{i=1}^{n}[\frac{(2i-1)\log(y_{\left(n-i-1\right)})\;y_{\left(n-i-1\right)}^{\kappa }\gamma ((\lambda -1)(1-\alpha )(1-z_{\left(n-i-1\right)}{)}^{2}+(2\lambda -2)(1-z_{\left(n-i-1\right)})-\lambda +\alpha +1)z_{\left(n-i-1\right)}}{((\lambda -1)(1-z_{\left(n-i-1\right)})-\lambda +1+\alpha )(1+(\alpha -1)(1-z_{\left(n-i-1\right)}))z_{\left(n-i-1\right)}}]=0$$

$$\frac{\partial ADE(y_{i})}{\partial \gamma }=\sum_{i=1}^{n}[\frac{(2i-1)((\lambda -1)(\alpha -1)z_{\left(i\right)}^{2}+(2\lambda -2)(1-z_{\left(i\right)})+\alpha +1-\lambda )\log(1-y_{\left(i\right)}^{\kappa })\;z_{\left(i\right)}}{((1-\lambda )(z_{\left(i\right)}-1)-1)(1+(\alpha -1)(1-z_{\left(i\right)}))(1-z_{\left(i\right)})z_{\left(i\right)}}]\;\;+\sum_{i=1}^{n}[\frac{(2i-1)((\lambda -1)(\alpha -1)z_{\left(i\right)}^{2}+(2\lambda -2)(1-z_{\left(i\right)})+\alpha +1-\lambda )\log(1-y_{\left(i\right)}^{\kappa })\;z_{\left(i\right)}}{\left((\lambda -1)z_{\left(i\right)}+\alpha +1-\lambda )(1+(\alpha -1)(1-z_{\left(i\right)})\right)}]=0$$

### 3.5 Method of Cramer-von Mises

Cramer-von Mises estimator (CvME) method is a minimization-based estimation method designed to determine the parameter that ensures the best fit between the observed data and the theoretical distribution. Accordingly, the CvME for the GMOKW distribution is obtained by minimizing the following function:

$$CvME\left(y_{i}\right)=\frac{1}{12n}+\sum_{i=1}^{n}{\left[\frac{\lambda \left(1-{\left(1-{y_{\left(i\right)}}^{\kappa }\right)}^{\gamma }\right)+\left(1-\lambda \right){\left(1-{\left(1-{y_{\left(i\right)}}^{\kappa }\right)}^{\gamma }\right)}^{2}}{\alpha +\left(1-\alpha \right)\left(1-{\left(1-{y_{\left(i\right)}}^{\kappa }\right)}^{\gamma }\right)}-\frac{2i-1}{2n}\right]}^{2}.$$ (13)

The minimization of the function given in Eq (13) is obtained by solving the system of equations obtained by taking the partial derivatives with respect to the parameters and setting them equal to zero. The partial derivatives of the function with respect to the parameters are given by


$$\frac{\partial CvME(y_{i})}{\partial \alpha }=\sum_{i=1}^{n}\left[\frac{\left(\frac{\lambda z_{\left(i\right)}+(1-\lambda )z_{\left(i\right)}^{2}}{\alpha +(1-\alpha )z_{\left(i\right)}}-\frac{2i-1}{2n}\right)\left[(\lambda z_{\left(i\right)}+(1-\lambda {)}^{2}z_{\left(i\right)}^{2})(1-z_{\left(i\right)})\right]}{{\left(\alpha +(1-\alpha )z_{\left(i\right)}\right)}^{2}}\right]=0,$$

$$\frac{\partial CvME(y_{i})}{\partial \lambda }=\sum_{i=1}^{n}\left[\frac{\left(\frac{\lambda z_{\left(i\right)}+(1-\lambda {)}^{2}z_{\left(i\right)}^{2}}{\alpha +(1-\alpha )z_{\left(i\right)}}-\frac{2i-1}{2n}\right)\left[z_{\left(i\right)}-2(1-\lambda )z_{\left(i\right)}^{2}\right]}{\alpha +(1-\alpha )z_{\left(i\right)}}\right]=0,$$

$$\frac{\partial LSE(y_{i})}{\partial \kappa }=\sum_{i=1}^{n}[w_{i}(\frac{\lambda (1-z_{\left(i\right)})\gamma y_{\left(i\right)}^{\kappa }\ln(y_{\left(i\right)})+2(1-\lambda {)}^{2}z_{\left(i\right)}(1-z_{\left(i\right)})\gamma y_{\left(i\right)}^{\kappa }\ln(y_{\left(i\right)})}{\left(1-y_{\left(i\right)}^{\kappa })(\alpha +(1-\alpha )z_{\left(i\right)}\right)}\;\;-\frac{\left(\lambda z_{\left(i\right)}+(1-\lambda {)}^{2}z_{\left(i\right)}^{2})(1-\alpha )(1-z_{\left(i\right)})\gamma y_{\left(i\right)}^{\kappa }\ln(y_{\left(i\right)}\right)}{(1-y_{\left(i\right)}^{\kappa })(\alpha +(1-\alpha )z_{\left(i\right)}{)}^{2}})\;\;\times (\frac{\lambda z_{\left(i\right)}+(1-\lambda {)}^{2}z_{\left(i\right)}^{2}}{\alpha +(1-\alpha )z_{\left(i\right)}}-\frac{2i-1}{2n})]=0,$$

$$\frac{\partial LSE(y_{i})}{\partial \gamma }=\sum_{i=1}^{n}[w_{i}(\frac{-\lambda (1-z_{\left(i\right)})\ln(1-y_{\left(i\right)}^{\kappa })-2(1-\lambda {)}^{2}(1-z_{\left(i\right)})z_{\left(i\right)}\ln(1-y_{\left(i\right)}^{\kappa })}{\alpha +(1-\alpha )z_{\left(i\right)}}\;\;+\frac{\left(\lambda z_{\left(i\right)}+(1-\lambda {)}^{2}z_{\left(i\right)}^{2})(1-\alpha )(1-z_{\left(i\right)})\ln(1-y_{\left(i\right)}^{\kappa }\right)}{(\alpha +(1-\alpha )z_{\left(i\right)}{)}^{2}})\;\;\times (\frac{\lambda z_{\left(i\right)}+(1-\lambda {)}^{2}z_{\left(i\right)}^{2}}{\alpha +(1-\alpha )z_{\left(i\right)}}-\frac{2i-1}{2n})]=0.$$

### 3.6 Method of maximum product spacing

The maximum product spacing estimator (MPSE) method is a method proposed by [29] as an alternative to the MLE. This method is based on maximizing the product of differences based on ordered observations. Accordingly, the MPSE of the parameter of the GMOKW distribution is obtained by maximizing the following function.


$$MPSE=\frac{1}{n+1}\sum_{i=1}^{n+1}\log\left(F\left(y_{\left(i\right)},\alpha ,\lambda ,\kappa ,\gamma \right)-F\left(y_{\left(i-1\right)},\alpha ,\lambda ,\kappa ,\gamma \right)\right)$$

where $F\left(y_{\left(0\right)},\alpha ,\lambda ,\kappa ,\gamma \right)=0$ and $F\left(y_{\left(n+1\right)},\alpha ,\lambda ,\kappa ,\gamma \right)=1$ and *F*(.) denote the cdf given in Eq (5). Accordingly, the MPSEs of the parameters are obtained by solving the following equations.


$$\frac{\partial MPSE}{\partial \alpha }=\frac{1}{n+1}\sum_{i=1}^{n+1}\left[\frac{F_{\alpha }^{\prime }\left(y_{\left(i\right)},\alpha ,\lambda ,\kappa ,\gamma \right)-F_{\alpha }^{\prime }\left(y_{\left(i-1\right)},\alpha ,\lambda ,\kappa ,\gamma \right)}{F\left(y_{\left(i\right)},\alpha ,\lambda ,\kappa ,\gamma \right)-F\left(y_{\left(i-1\right)},\alpha ,\lambda ,\kappa ,\gamma \right)}\right]=0,$$

$$\frac{\partial MPSE}{\partial \lambda }=\frac{1}{n+1}\left[\frac{F_{\lambda }^{\prime }\left(y_{\left(i\right)},\alpha ,\lambda ,\kappa ,\gamma \right)-F_{\lambda }^{\prime }\left(y_{\left(i-1\right)},\alpha ,\lambda ,\kappa ,\gamma \right)}{F\left(y_{\left(i\right)},\alpha ,\lambda ,\kappa ,\gamma \right)-F\left(y_{\left(i-1\right)},\alpha ,\lambda ,\kappa ,\gamma \right)}\right]=0,$$

$$\frac{\partial MPSE}{\partial \kappa }=\frac{1}{n+1}\left[\frac{F_{\kappa }^{\prime }\left(y_{\left(i\right)},\alpha ,\lambda ,\kappa ,\gamma \right)-F_{\kappa }^{\prime }\left(y_{\left(i-1\right)},\alpha ,\lambda ,\kappa ,\gamma \right)}{F\left(y_{\left(i\right)},\alpha ,\lambda ,\kappa ,\gamma \right)-F\left(y_{\left(i-1\right)},\alpha ,\lambda ,\kappa ,\gamma \right)}\right]=0,$$

$$\frac{\partial MPSE}{\partial \gamma }=\frac{1}{n+1}\left[\frac{F_{\gamma }^{\prime }\left(y_{\left(i\right)},\alpha ,\lambda ,\kappa ,\gamma \right)-F_{\gamma }^{\prime }\left(y_{\left(i-1\right)},\alpha ,\lambda ,\kappa ,\gamma \right)}{F\left(y_{\left(i\right)},\alpha ,\lambda ,\kappa ,\gamma \right)-F\left(y_{\left(i-1\right)},\alpha ,\lambda ,\kappa ,\gamma \right)}\right]=0.$$

The terms $F_{\alpha }^{\prime }(\cdot )$, $F_{\lambda }^{\prime }(\cdot )$, $F_{\kappa }^{\prime }(\cdot )$, and $F_{\gamma }^{\prime }(\cdot )$ are respectively defined as follows.

$$F_{\alpha }^{\prime }(\cdot )=-\frac{[\lambda \left(1-(1-y^{\kappa }{)}^{\gamma }\right)+(1-\lambda ){\left(1-(1-y^{\kappa }{)}^{\gamma }\right)}^{2}{]}^{\gamma }(1-y^{\kappa }{)}^{\gamma }}{[\alpha +(1-\alpha )\left(1-(1-y^{\kappa }{)}^{\gamma }\right){]}^{2}},$$ (14)

$$F_{\lambda }^{\prime }(\cdot )=\frac{\left[1-(1-y^{\kappa }{)}^{\gamma }\right]-{\left[1-(1-y^{\kappa }{)}^{\gamma }\right]}^{2}}{\alpha +(1-\alpha )\left[1-(1-y^{\kappa }{)}^{\gamma }\right]},$$ (15)

$$F_{\kappa }^{\prime }(\cdot )=-\frac{\ln(y)\;y^{\kappa }\;\gamma (1-y^{\kappa }{)}^{\gamma -1}[(1-\lambda )(1-\alpha ){\left(1-(1-y^{\kappa }{)}^{\gamma }\right)}^{2}+(2\lambda -2)(1-y^{\kappa }{)}^{\gamma }-\lambda +1+\alpha ]}{(1-y^{\kappa })[1+(1-\alpha )(1-y^{\kappa }{)}^{\gamma }{]}^{2}},$$ (16)

$$F_{\gamma }^{\prime }(\cdot )=-\frac{\ln(1-y^{\kappa })\;(1-y^{\kappa }{)}^{\gamma }[(1-\lambda )(1-\alpha ){\left(1-(1-y^{\kappa }{)}^{\gamma }\right)}^{2}+(2\lambda -2)(1-y^{\kappa }{)}^{\gamma }-\lambda +1+\alpha ]}{[1+(1-\alpha )(1-y^{\kappa }{)}^{\gamma }{]}^{2}}$$ (17)

### 3.7 Method of right tail Anderson-Darling

This estimation method aims to minimize the Anderson-Darling statistic in the right tail to estimate the parameters of a specific distribution. Accordingly, the right tail Anderson-Darling estimator (RTADE) for the GMOKW distribution is obtained by minimizing the following objective function.

$$CvME\left(y_{i}\right)=\frac{n}{2}-2\sum_{i=1}^{n}F\left(y_{\left(i\right)},\alpha ,\lambda ,\kappa ,\gamma \right)-\frac{1}{n}\sum_{i=1}^{n}\left(2i-1\right)\log\left(1-F\left(y_{\left(n-i-1\right)},\alpha ,\lambda ,\kappa ,\gamma \right)\right),$$ (18)

where *F*(.) represents cdf defined in Eq (5). The RTADEs of the parameters *α*, *λ*, κ, and *γ* are obtained by solving the following system of equations.


$$\frac{\partial CvME(y_{i})}{\partial \alpha }=-2\sum_{i=1}^{n}F_{\alpha }^{\prime }(y_{\left(i\right)};\alpha ,\lambda ,\kappa ,\gamma )+\frac{1}{n}\sum_{i=1}^{n}(2i-1)\frac{F_{\alpha }^{\prime }(y_{\left(n-i-1\right)};\alpha ,\lambda ,\kappa ,\gamma )}{1-F(y_{\left(n-i-1\right)};\alpha ,\lambda ,\kappa ,\gamma )}=0,$$

$$\frac{\partial CvME(y_{i})}{\partial \lambda }=-2\sum_{i=1}^{n}F_{\lambda }^{\prime }(y_{\left(i\right)};\alpha ,\lambda ,\kappa ,\gamma )+\frac{1}{n}\sum_{i=1}^{n}(2i-1)\frac{F_{\lambda }^{\prime }(y_{\left(n-i-1\right)};\alpha ,\lambda ,\kappa ,\gamma )}{1-F(y_{\left(n-i-1\right)};\alpha ,\lambda ,\kappa ,\gamma )}=0,$$

$$\frac{\partial CvME(y_{i})}{\partial \kappa }=-2\sum_{i=1}^{n}F_{\kappa }^{\prime }(y_{\left(i\right)};\alpha ,\lambda ,\kappa ,\gamma )+\frac{1}{n}\sum_{i=1}^{n}(2i-1)\frac{F_{\kappa }^{\prime }(y_{\left(n-i-1\right)};\alpha ,\lambda ,\kappa ,\gamma )}{1-F(y_{\left(n-i-1\right)};\alpha ,\lambda ,\kappa ,\gamma )}=0,$$

$$\frac{\partial CvME(y_{i})}{\partial \gamma }=-2\sum_{i=1}^{n}F_{\gamma }^{\prime }(y_{\left(i\right)};\alpha ,\lambda ,\kappa ,\gamma )+\frac{1}{n}\sum_{i=1}^{n}(2i-1)\frac{F_{\gamma }^{\prime }(y_{\left(n-i-1\right)};\alpha ,\lambda ,\kappa ,\gamma )}{1-F(y_{\left(n-i-1\right)};\alpha ,\lambda ,\kappa ,\gamma )}=0,$$

where $F_{\alpha }^{\prime }\left(.\right),F_{\lambda }^{\prime }\left(.\right),F_{\kappa }^{\prime }\left(.\right)$ and $F_{\alpha }^{\prime }\left(.\right)$ are given in Eqs (14)–(17).

### 3.8 Method of minimum spacing absolute distance

The minimum spacing absolute distance estimator (MSADE) is obtained by minimizing the absolute difference between the ordered observations. Thus, the MSADE for the GMOKW distribution is obtained by minimizing the following function.

$$\Psi \left(y_{i}\right)=\sum_{i=1}^{n+1}\left|F\left(y_{\left(i\right)},\alpha ,\lambda ,\kappa ,\gamma \right)-F\left(y_{\left(i-1\right)},\alpha ,\lambda ,\kappa ,\gamma \right)\right|$$ (19)

where *F*(.) denote the cdf given in Eq (5). The MSADEs of the parameters *α*, *λ*, κ, and *γ* are obtained by minimizing the function in Eq (19) with respect to these parameters. This requires solving the following system of equations:


$$\frac{\partial \Psi (y_{i})}{\partial \alpha }=\sum_{i=1}^{n+1}\text{sign}(g_{i})\left[F_{\alpha }^{\prime }(y_{\left(i\right)};\alpha ,\lambda ,\kappa ,\gamma )-F_{\alpha }^{\prime }(y_{\left(i-1\right)};\alpha ,\lambda ,\kappa ,\gamma )\right]=0,$$

$$\frac{\partial \Psi (y_{i})}{\partial \lambda }=\sum_{i=1}^{n+1}\text{sign}(g_{i})\left[F_{\lambda }^{\prime }(y_{\left(i\right)};\alpha ,\lambda ,\kappa ,\gamma )-F_{\lambda }^{\prime }(y_{\left(i-1\right)};\alpha ,\lambda ,\kappa ,\gamma )\right]=0,$$

$$\frac{\partial \Psi (y_{i})}{\partial \kappa }=\sum_{i=1}^{n+1}\text{sign}(g_{i})\left[F_{\kappa }^{\prime }(y_{\left(i\right)};\alpha ,\lambda ,\kappa ,\gamma )-F_{\kappa }^{\prime }(y_{\left(i-1\right)};\alpha ,\lambda ,\kappa ,\gamma )\right]=0,$$

$$\frac{\partial \Psi (y_{i})}{\partial \gamma }=\sum_{i=1}^{n+1}\text{sign}(g_{i})\left[F_{\gamma }^{\prime }(y_{\left(i\right)};\alpha ,\lambda ,\kappa ,\gamma )-F_{\gamma }^{\prime }(y_{\left(i-1\right)};\alpha ,\lambda ,\kappa ,\gamma )\right]=0,$$

where $g_{i}=\left[F(y_{\left(i\right)};\alpha ,\lambda ,\kappa ,\gamma )-F(y_{\left(i-1\right)};\alpha ,\lambda ,\kappa ,\gamma )-\frac{1}{n+1}\right]$ and $\text{sign}(g_{i})$ demonstrate the sign function defined as


$$\text{sign}(g_{i})=\left\{ \begin{array}{cc} +1, & \text{if }g_{i}>0,\\ -1, & \text{if }g_{i}<0. \end{array}\right.$$

Moreover the functions $F_{\alpha }^{\prime }\left(.\right),F_{\lambda }^{\prime }\left(.\right),F_{\kappa }^{\prime }\left(.\right)$ and $F_{\alpha }^{\prime }\left(.\right)$ are given in Eqs (14)–(17).

### 3.9 Method of minimum spacing absolute-log distance

The minimum spacing absolute-log distance estimator (MSALDE) is obtained by minimizing the absolute logarithm difference between the ordered observations. Thus, the MSALDE for the GMOKW distribution is obtained by minimizing the following function:

$$\psi_{\ell }=\sum_{i=1}^{n+1}\left|\log\left(F\left(y_{\left(i\right)},\alpha ,\lambda ,\kappa ,\gamma \right)-F\left(y_{\left(i-1\right)},\alpha ,\lambda ,\kappa ,\gamma \right)\right)-\log\left(\frac{1}{n+1}\right)\right|$$ (20)

where *F*(.) demosrate the cdf given in Eq (5). The MSALDEs of the parameters *α*, *λ*, κ, and *γ* are obtained by minimizing the function in Eq (20) with respect to these parameters.


$$\frac{\partial \Psi_{\ell }}{\partial \alpha }=\sum_{i=1}^{n+1}\text{sign}(g_{i})\left(\frac{F_{\alpha }^{\prime }(y_{\left(i\right)};\alpha ,\lambda ,\kappa ,\gamma )-F_{\alpha }^{\prime }(y_{\left(i-1\right)};\alpha ,\lambda ,\kappa ,\gamma )}{F(y_{\left(i\right)};\alpha ,\lambda ,\kappa ,\gamma )-F(y_{\left(i-1\right)};\alpha ,\lambda ,\kappa ,\gamma )}\right)=0,$$

$$\frac{\partial \Psi_{\ell }}{\partial \lambda }=\sum_{i=1}^{n+1}\text{sign}(g_{i})\left(\frac{F_{\lambda }^{\prime }(y_{\left(i\right)};\alpha ,\lambda ,\kappa ,\gamma )-F_{\lambda }^{\prime }(y_{\left(i-1\right)};\alpha ,\lambda ,\kappa ,\gamma )}{F(y_{\left(i\right)};\alpha ,\lambda ,\kappa ,\gamma )-F(y_{\left(i-1\right)};\alpha ,\lambda ,\kappa ,\gamma )}\right)=0,$$

$$\frac{\partial \Psi_{\ell }}{\partial \kappa }=\sum_{i=1}^{n+1}\text{sign}(g_{i})\left(\frac{F_{\kappa }^{\prime }(y_{\left(i\right)};\alpha ,\lambda ,\kappa ,\gamma )-F_{\kappa }^{\prime }(y_{\left(i-1\right)};\alpha ,\lambda ,\kappa ,\gamma )}{F(y_{\left(i\right)};\alpha ,\lambda ,\kappa ,\gamma )-F(y_{\left(i-1\right)};\alpha ,\lambda ,\kappa ,\gamma )}\right)=0,$$

$$\frac{\partial \Psi_{\ell }}{\partial \gamma }=\sum_{i=1}^{n+1}\text{sign}(g_{i})\left(\frac{F_{\gamma }^{\prime }(y_{\left(i\right)};\alpha ,\lambda ,\kappa ,\gamma )-F_{\gamma }^{\prime }(y_{\left(i-1\right)};\alpha ,\lambda ,\kappa ,\gamma )}{F(y_{\left(i\right)};\alpha ,\lambda ,\kappa ,\gamma )-F(y_{\left(i-1\right)};\alpha ,\lambda ,\kappa ,\gamma )}\right)=0,$$

where $g_{i}=\log\;\left(F(y_{\left(i\right)};\alpha ,\lambda ,\kappa ,\gamma )-F(y_{\left(i-1\right)};\alpha ,\lambda ,\kappa ,\gamma )\right)-\log\;\left(\frac{1}{n+1}\right)$ and $\text{sign}(g_{i})$ demonstrate the sign function defined as


$$\text{sign}(g_{i})=\left\{ \begin{array}{cc} +1, & \text{if }g_{i}>0,\\ -1, & \text{if }g_{i}<0. \end{array}\right.$$

Moreover, the functions $F_{\alpha }^{\prime }\left(.\right),F_{\lambda }^{\prime }\left(.\right),F_{\kappa }^{\prime }\left(.\right)$ and $F_{\alpha }^{\prime }\left(.\right)$ are given in Eqs (14)–(17).

Since the systems of equations derived from all minimization and maximization process involve nonlinear expressions, the parameter estimators cannot be obtained in closed form. Therefore, numerical optimization algorithms such as BFGS, Nelder–Mead, CG, and L-BFGS-B implemented in the R software are used to obtain the parameter estimators.

## 4 Simulation study

In this section, a Monte Carlo simulation study is conducted to evaluate the performance of the parameter estimators for the GMOKW distribution based on MLE, LSE, WLSE, ADE, CvM, MPSE, RTADE,MSADE, and MSALDE methods.The simulation study is carried out following these steps

- The simulation study is conducted with *N* = 1000 replications, and the parameters values are settings $Case_{1}=(\alpha =0.25,\lambda =0.5,\kappa =1.2,\gamma =1.5)$, $Case_{2}=(\alpha =0.2,\lambda =0.7,\kappa =0.5,\gamma =0.75)$ and $Case_{3}=(\alpha =0.25,\lambda =0.75,\kappa =2,\gamma =3)$.
- Random numbers are generated from the GMOKW distribution for the specified parameter values with size of n=50,100,200,500,750 and 1000.
- $\left({\hat{\alpha }}_{i},{\hat{\lambda }}_{i},{\hat{\kappa }}_{i},{\hat{\gamma }}_{i}\right)$ estimates are obtained for each parameter using the generated samples for i=(1,2,...,1000).
- The performance of the estimators is evaluated in terms of bias and mean squared errors (MSEs) using the estimated values $\hat{\phi }=\left(\hat{\alpha },\hat{\lambda },\hat{\kappa },\hat{\gamma }\right)$ and the true parameter value ϕ=(α,λ,κ,γ). The bias and MSEs are obtained by calculating the following equations.

$$Bias_{\phi }=\frac{1}{N}\sum_{i=1}^{N}(\hat{𝜙\phi_{i}}-𝜙\phi )$$

$$MSE_{\phi }=\frac{1}{N}\sum_{i=1}^{N}(\hat{𝜙\phi_{i}}-𝜙\phi {)}^{2}.$$

**Fig 3:**
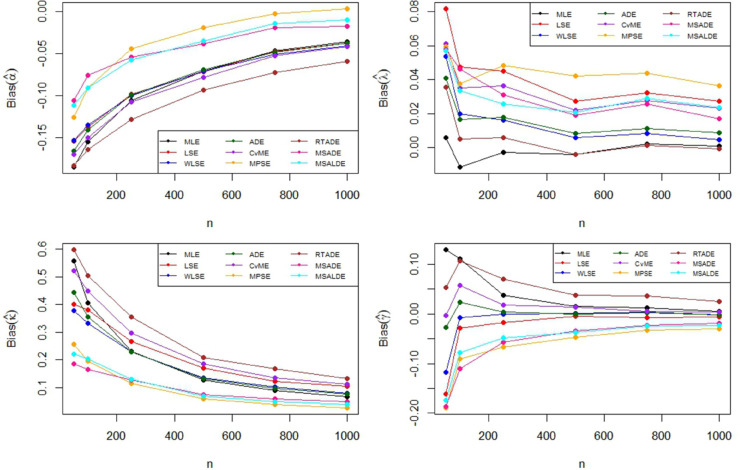
Graphical representation of bias values in Table 2.

**Fig 4:**
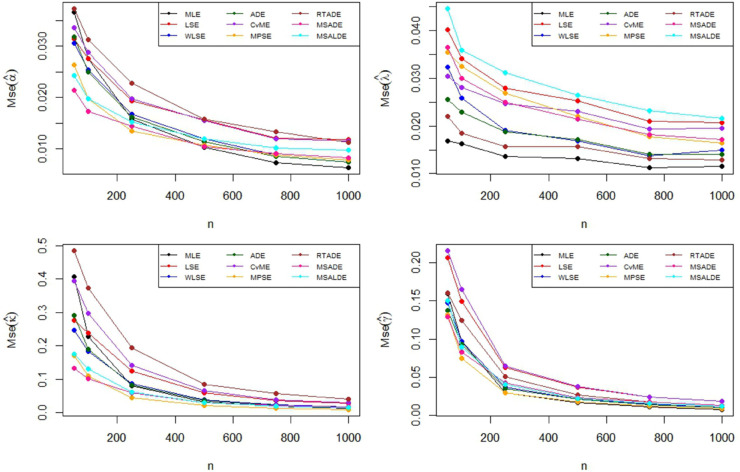
Graphical representation of MSE values in Table 2.

**Fig 5:**
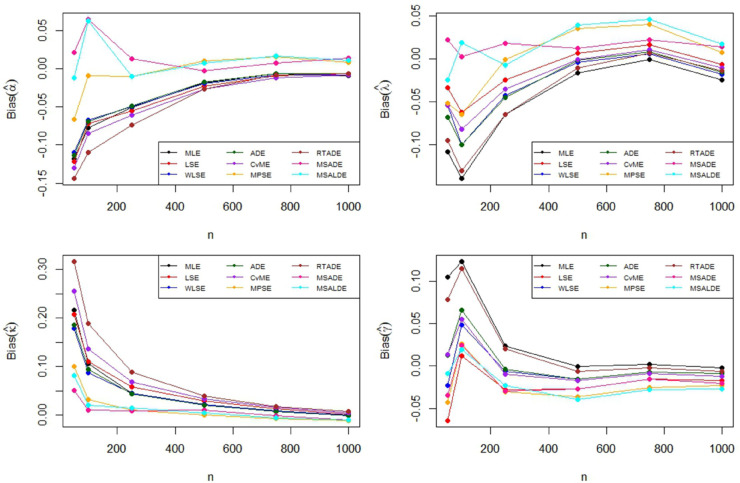
Graphical representation of bias values in Table 3.

**Fig 6:**
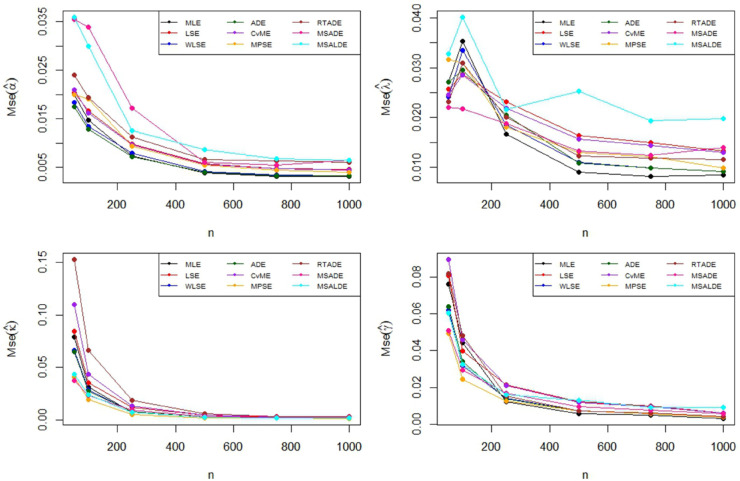
Graphical representation of MSE values in Table 3.

**Fig 7:**
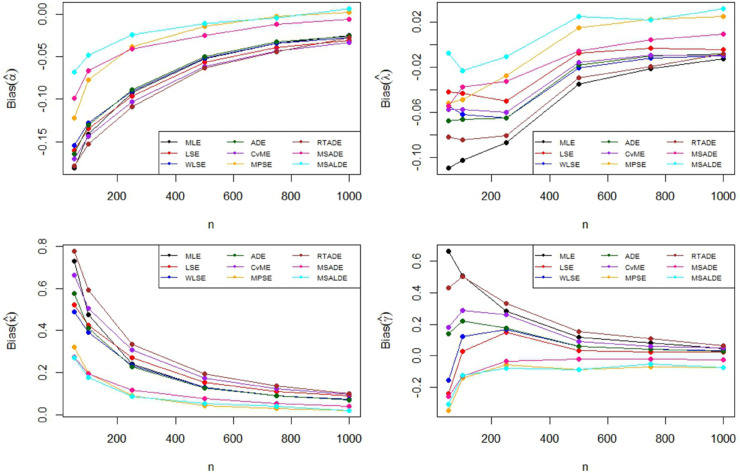
Graphical representation of bias values in Table 4.

**Fig 8:**
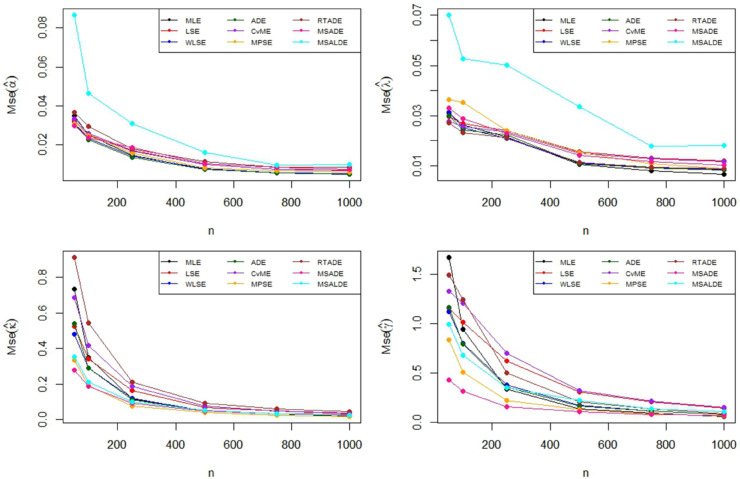
Graphical representation of MSE values in Table 4.

The Monte Carlo simulation steps for parameter estimation are summarized in Table 2. Simulation results are reported in Tables 3–5 and their graphical representation are given in Figs 3–8. The simulation results presented in Tables 3–5 can be summarized as follows.

**Table 2:** Monte Carlo simulation steps for parameter estimation.

**Step I:** Set the true parameter values ϕ=(α,λ,κ,γ) and the sample sizes (n=50,100,250,500,750,1000).
**Step II:** For each sample size, repeat the following steps *N* = 1000 times:
i. Generate random samples from the GMOKW distribution using the quantile function defined in Eq (7). That is, generate $U_{i}\sim \text{Uniform}(0,1)$ for i=1,2,…,n and compute $x_{i}=Q(U_{i};\alpha ,\lambda ,\kappa ,\gamma )$.
ii. Estimate the parameters using the MLE, LSE, WLSE, ADE, CvME, MPSE, RTADE, MSADE, and MSALDE methods.
iii. Compute the bias and MSE values for each estimation method.

**Table 3:** The bias and MSEs of all estimators for unknown parameters in *Case*_1_.

n	Measure	Methods	MLE	LSE	WLSE	ADE	CvME	MPSE	RTADE	MSADE	MSALDE
50	Bias	$\hat{\alpha }$	–0.1855	–0.1544	–0.1539	–0.1657	–0.1701	–0.1260	–0.1833	-0.1056	–0.1117
		$\hat{\lambda }$	0.0059	0.0816	0.0539	0.0411	0.0610	0.0594	0.0358	0.0577	0.0571
		$\hat{\kappa }$	0.5559	0.3996	0.3777	0.4442	0.5214	0.2569	0.5977	0.1841	0.2207
		$\hat{\gamma }$	0.1296	–0.1606	–0.1173	–0.0272	–0.0026	–0.1892	0.0536	-0.1873	–0.1733
	MSE	$\hat{\alpha }$	0.0366	0.0314	0.0306	0.0318	0.0336	0.0263	0.0372	0.0214	0.0243
		$\hat{\lambda }$	0.0168	0.0402	0.0323	0.0256	0.0304	0.0354	0.0220	0.0365	0.0445
		$\hat{\kappa }$	0.4075	0.2762	0.2462	0.2910	0.3939	0.1702	0.4844	0.1338	0.1748
		$\hat{\gamma }$	0.1595	0.2065	0.1479	0.1370	0.2155	0.1322	0.1603	0.1289	0.1504
100	Bias	$\hat{\alpha }$	–0.1554	–0.1381	–0.1354	–0.1408	–0.1500	–0.0912	–0.1646	-0.0759	–0.0911
		$\hat{\lambda }$	–0.0115	0.0477	0.0201	0.0168	0.0352	0.0376	0.0051	0.0465	0.0336
		$\hat{\kappa }$	0.4051	0.3799	0.3327	0.3549	0.4490	0.1959	0.5044	0.1641	0.2029
		$\hat{\gamma }$	0.1111	–0.0285	–0.0074	0.0238	0.0573	–0.0911	0.1073	-0.1111	–0.0785
	MSE	$\hat{\alpha }$	0.0276	0.0276	0.0253	0.0250	0.0288	0.0197	0.0313	0.0173	0.0197
		$\hat{\lambda }$	0.0162	0.0341	0.0259	0.0229	0.0281	0.0325	0.0185	0.0299	0.0359
		$\hat{\kappa }$	0.2285	0.2384	0.1842	0.1905	0.2981	0.1106	0.3726	0.1012	0.1301
		$\hat{\gamma }$	0.0967	0.1492	0.0970	0.0934	0.1647	0.0747	0.1246	0.0836	0.0895
250	Bias	$\hat{\alpha }$	–0.1057	–0.0987	–0.1002	–0.0993	–0.1076	–0.0442	–0.1282	–0.0546	–0.0579
		$\hat{\lambda }$	–0.0026	0.0450	0.0164	0.0180	0.0365	0.0485	0.0058	0.0310	0.0257
		$\hat{\kappa }$	0.2307	0.2661	0.2280	0.2278	0.2963	0.1140	0.3536	0.1279	0.1306
		$\hat{\gamma }$	0.0384	–0.0172	0.0001	0.0045	0.0175	–0.0669	0.0701	–0.0569	–0.0481
	MSE	$\hat{\alpha }$	0.0158	0.0194	0.0167	0.0162	0.0197	0.0134	0.0228	0.0144	0.0153
		$\hat{\lambda }$	0.0136	0.0279	0.0191	0.0187	0.0246	0.0269	0.0156	0.0249	0.0311
		$\hat{\kappa }$	0.0807	0.1241	0.0867	0.0820	0.1415	0.0452	0.1950	0.0605	0.0606
		$\hat{\gamma }$	0.0300	0.0631	0.0370	0.0353	0.0650	0.0300	0.0508	0.0424	0.0408
500	Bias	$\hat{\alpha }$	–0.0705	–0.0718	–0.0713	–0.0690	–0.0781	–0.0194	–0.0938	–0.0387	–0.0350
		$\hat{\lambda }$	–0.0040	0.0275	0.0060	0.0085	0.0220	0.0421	–0.0040	0.0190	0.0206
		$\hat{\kappa }$	0.1269	0.1693	0.1356	0.1329	0.1852	0.0577	0.2082	0.0747	0.0694
		$\hat{\gamma }$	0.0149	–0.0039	0.0004	0.0003	0.0136	–0.0473	0.0380	–0.0338	–0.0372
	MSE	$\hat{\alpha }$	0.0103	0.0156	0.0119	0.0114	0.0155	0.0107	0.0158	0.0104	0.0120
		$\hat{\lambda }$	0.0132	0.0252	0.0169	0.0171	0.0231	0.0220	0.0156	0.0214	0.0265
		$\hat{\kappa }$	0.0325	0.0595	0.0376	0.0356	0.0652	0.0208	0.0843	0.0297	0.0296
		$\hat{\gamma }$	0.0171	0.0374	0.0220	0.0217	0.0380	0.0182	0.0273	0.0236	0.0229
750	Bias	$\hat{\alpha }$	–0.0471	–0.0472	–0.0511	–0.0483	–0.0528	–0.0021	–0.0725	–0.0195	-0.0140
		$\hat{\lambda }$	0.0023	0.0325	0.0085	0.0113	0.0277	0.0440	0.0016	0.0258	0.0291
		$\hat{\kappa }$	0.0890	0.1231	0.1011	0.0978	0.1341	0.0383	0.1679	0.0577	0.0491
		$\hat{\gamma }$	0.0122	–0.0067	0.0042	0.0030	0.0049	–0.0334	0.0365	–0.0228	–0.0241
	MSE	$\hat{\alpha }$	0.0073	0.0121	0.0088	0.0085	0.0119	0.0088	0.0133	0.0091	0.0102
		$\hat{\lambda }$	0.0112	0.0210	0.0137	0.0140	0.0194	0.0177	0.0132	0.0181	0.0232
		$\hat{\kappa }$	0.0198	0.0359	0.0240	0.0223	0.0388	0.0136	0.0577	0.0227	0.0205
		$\hat{\gamma }$	0.0114	0.0244	0.0149	0.0145	0.0246	0.0120	0.0183	0.0177	0.0165
1000	Bias	$\hat{\alpha }$	–0.0358	–0.0373	–0.0409	–0.0374	–0.0421	0.0030	–0.0595	–0.0173	–0.0101
		$\hat{\lambda }$	0.0009	0.0273	0.0047	0.0090	0.0233	0.0364	–0.0008	0.0170	0.0238
		$\hat{\kappa }$	0.0674	0.1034	0.0780	0.0765	0.1119	0.0265	0.1328	0.0475	0.0398
		$\hat{\gamma }$	0.0058	–0.0053	–0.0011	–0.0018	0.0036	–0.0301	0.0248	–0.0182	-0.0224
	MSE	$\hat{\alpha }$	0.0063	0.0118	0.0079	0.0074	0.0115	0.0078	0.0112	0.0083	0.0097
		$\hat{\lambda }$	0.0115	0.0207	0.0149	0.0140	0.0195	0.0164	0.0129	0.0172	0.0216
		$\hat{\kappa }$	0.0132	0.0281	0.0169	0.0155	0.0299	0.0095	0.0403	0.0165	0.0147
		$\hat{\gamma }$	0.0080	0.0188	0.0108	0.0106	0.0189	0.0087	0.0129	0.0129	0.0121

**Table 4:** The bias and MSEs of all estimators for unknown parameters in *Case*_2_.

n	Measure	Methods	MLE	LSE	WLSE	ADE	CvME	MPSE	RTADE	MSADE	MSALDE
50	Bias	$\hat{\alpha }$	–0.1184	–0.1217	–0.1101	–0.1134	–0.1303	–0.0663	–0.1442	0.0211	–0.0124
		$\hat{\lambda }$	–0.1082	–0.0339	–0.0543	–0.0676	–0.0544	–0.0519	–0.0946	0.0222	–0.0242
		$\hat{\kappa }$	0.2163	0.2073	0.1789	0.1861	0.2560	0.1000	0.3165	0.0515	0.0814
		$\hat{\gamma }$	0.1054	–0.0643	–0.0232	0.0127	0.0133	–0.0425	0.0789	–0.0347	–0.0088
	MSE	$\hat{\alpha }$	0.0199	0.0203	0.0184	0.0175	0.0210	0.0199	0.0240	0.0355	0.0358
		$\hat{\lambda }$	0.0271	0.0257	0.0241	0.0271	0.0246	0.0317	0.0232	0.0220	0.0328
		$\hat{\kappa }$	0.0789	0.0843	0.0663	0.0649	0.1100	0.0408	0.1530	0.0375	0.0433
		$\hat{\gamma }$	0.0759	0.0805	0.0617	0.0638	0.0894	0.0494	0.0819	0.0507	0.0604
100	Bias	$\hat{\alpha }$	–0.0772	–0.0722	–0.0671	–0.0690	–0.0850	–0.0096	–0.1096	0.0641	0.0624
		$\hat{\lambda }$	–0.1392	–0.0621	–0.0999	–0.1000	–0.0817	–0.0645	–0.1296	0.0020	0.0184
		$\hat{\kappa }$	0.1056	0.1097	0.0878	0.0938	0.1366	0.0316	0.1881	0.0099	0.0206
		$\hat{\gamma }$	0.1235	0.0123	0.0488	0.0658	0.0549	0.0264	0.1153	0.0249	0.0194
	MSE	$\hat{\alpha }$	0.0147	0.0166	0.0134	0.0128	0.0162	0.0191	0.0193	0.0338	0.0300
		$\hat{\lambda }$	0.0353	0.0287	0.0335	0.0295	0.0286	0.0310	0.0310	0.0217	0.0401
		$\hat{\kappa }$	0.0318	0.0356	0.0289	0.0275	0.0435	0.0194	0.0662	0.0233	0.0240
		$\hat{\gamma }$	0.0444	0.0396	0.0318	0.0338	0.0459	0.0244	0.0482	0.0292	0.0323
250	Bias	$\hat{\alpha }$	–0.0504	–0.0554	–0.0497	–0.0493	–0.0612	–0.0106	–0.0735	0.0131	–0.0103
		$\hat{\lambda }$	–0.0644	–0.0249	–0.0426	–0.0449	–0.0353	–0.0010	–0.0648	0.0177	–0.0075
		$\hat{\kappa }$	0.0455	0.0579	0.0450	0.0431	0.0685	0.0105	0.0893	0.0088	0.0145
		$\hat{\gamma }$	0.0237	–0.0279	–0.0064	–0.0034	–0.0097	–0.0305	0.0201	–0.0296	–0.0226
	MSE	$\hat{\alpha }$	0.0074	0.0098	0.0079	0.0072	0.0097	0.0093	0.0112	0.0172	0.0126
		$\hat{\lambda }$	0.0167	0.0231	0.0185	0.0205	0.0219	0.0181	0.0200	0.0187	0.0216
		$\hat{\kappa }$	0.0075	0.0121	0.0081	0.0078	0.0135	0.0055	0.0184	0.0094	0.0071
		$\hat{\gamma }$	0.0120	0.0213	0.0140	0.0136	0.0208	0.0126	0.0153	0.0169	0.0160
500	Bias	$\hat{\alpha }$	–0.0186	–0.0230	–0.0191	–0.0176	–0.0267	0.0097	–0.0268	–0.0030	0.0076
		$\hat{\lambda }$	–0.0162	0.0068	–0.0042	–0.0018	–0.0007	0.0347	–0.0103	0.0119	0.0392
		$\hat{\kappa }$	0.0210	0.0286	0.0214	0.0209	0.0338	0.0004	0.0388	0.0099	0.0042
		$\hat{\gamma }$	–0.0002	–0.0273	–0.0157	–0.0151	–0.0174	–0.0365	–0.0063	–0.0270	–0.0395
	MSE	$\hat{\alpha }$	0.0039	0.0057	0.0041	0.0040	0.0056	0.0054	0.0066	0.0060	0.0087
		$\hat{\lambda }$	0.0090	0.0164	0.0110	0.0108	0.0157	0.0129	0.0123	0.0133	0.0253
		$\hat{\kappa }$	0.0025	0.0045	0.0028	0.0026	0.0048	0.0020	0.0061	0.0033	0.0028
		$\hat{\gamma }$	0.0058	0.0122	0.0071	0.0071	0.0118	0.0074	0.0074	0.0097	0.0128
750	Bias	$\hat{\alpha }$	–0.0066	–0.0091	–0.0079	–0.0064	–0.0118	0.0156	–0.0086	0.0072	0.0169
		$\hat{\lambda }$	–0.0007	0.0159	0.0056	0.0080	0.0103	0.0403	0.0068	0.0218	0.0453
		$\hat{\kappa }$	0.0075	0.0126	0.0083	0.0078	0.0160	–0.0074	0.0180	–0.0007	–0.0058
		$\hat{\gamma }$	0.0022	–0.0156	–0.0067	–0.0067	–0.0087	–0.0257	–0.0021	–0.0155	–0.0276
	MSE	$\hat{\alpha }$	0.0032	0.0047	0.0034	0.0033	0.0047	0.0044	0.0063	0.0054	0.0067
		$\hat{\lambda }$	0.0081	0.0150	0.0098	0.0098	0.0144	0.0121	0.0118	0.0124	0.0193
		$\hat{\kappa }$	0.0016	0.0029	0.0018	0.0017	0.0031	0.0016	0.0036	0.0024	0.0022
		$\hat{\gamma }$	0.0048	0.0099	0.0061	0.0060	0.0097	0.0057	0.0059	0.0075	0.0093
1000	Bias	$\hat{\alpha }$	–0.0092	–0.0064	–0.0081	–0.0066	–0.0087	0.0087	–0.0069	0.0140	0.0106
		$\hat{\lambda }$	–0.0246	–0.0067	–0.0184	–0.0160	–0.0110	0.0070	–0.0140	0.0134	0.0167
		$\hat{\kappa }$	0.0005	0.0016	–0.0004	–0.0009	0.0041	–0.0112	0.0071	–0.0097	–0.0106
		$\hat{\gamma }$	–0.0019	–0.0170	–0.0086	–0.0089	–0.0121	–0.0225	–0.0061	–0.0201	–0.0270
	MSE	$\hat{\alpha }$	0.0031	0.0046	0.0033	0.0033	0.0045	0.0040	0.0061	0.0065	0.0064
		$\hat{\lambda }$	0.0084	0.0132	0.0091	0.0091	0.0129	0.0099	0.0116	0.0140	0.0197
		$\hat{\kappa }$	0.0014	0.0023	0.0015	0.0015	0.0023	0.0015	0.0030	0.0024	0.0022
		$\hat{\gamma }$	0.0031	0.0061	0.0037	0.0037	0.0059	0.0039	0.0040	0.0058	0.0090

**Table 5:** The bias and MSEs of all estimators for unknown parameters in *Case*_3_.

n	Measure	Methods	MLE	LSE	WLSE	ADE	CvME	MPSE	RTADE	MSADE	MSALDE
50	Bias	$\hat{\alpha }$	–0.1813	–0.1605	–0.1548	–0.1642	–0.1702	–0.1223	–0.1788	–0.0991	–0.0683
		$\hat{\lambda }$	–0.1093	–0.0415	–0.0540	–0.0675	–0.0573	–0.0518	–0.0816	–0.0544	–0.0073
		$\hat{\kappa }$	0.7294	0.5221	0.4896	0.5737	0.6627	0.3195	0.7759	0.2733	0.2712
		$\hat{\gamma }$	0.6646	–0.2411	–0.1542	0.1394	0.1780	–0.3478	0.4301	–0.2600	–0.3083
	MSE	$\hat{\alpha }$	0.0351	0.0323	0.0314	0.0309	0.0334	0.0310	0.0366	0.0297	0.0865
		$\hat{\lambda }$	0.0310	0.0296	0.0314	0.0299	0.0276	0.0363	0.0271	0.0329	0.0700
		$\hat{\kappa }$	0.7334	0.5237	0.4789	0.5414	0.6857	0.3333	0.9129	0.2784	0.3531
		$\hat{\gamma }$	1.6682	1.1539	1.1196	1.1636	1.3278	0.8326	1.4935	0.4309	0.9906
100	Bias	$\hat{\alpha }$	–0.1412	–0.1342	–0.1283	–0.1307	–0.1434	–0.0771	–0.1525	–0.0663	–0.0479
		$\hat{\lambda }$	–0.1025	–0.0428	–0.0620	–0.0658	–0.0575	–0.0485	–0.0842	–0.0371	–0.0231
		$\hat{\kappa }$	0.4761	0.4232	0.3901	0.4102	0.5054	0.1967	0.5912	0.1925	0.1757
		$\hat{\gamma }$	0.5080	0.0290	0.1229	0.2189	0.2862	–0.1420	0.5036	–0.1302	–0.1242
	MSE	$\hat{\alpha }$	0.0250	0.0250	0.0233	0.0228	0.0258	0.0247	0.0294	0.0238	0.0466
		$\hat{\lambda }$	0.0262	0.0268	0.0250	0.0244	0.0257	0.0352	0.0231	0.0288	0.0528
		$\hat{\kappa }$	0.3495	0.3415	0.2900	0.2897	0.4176	0.1922	0.5433	0.1874	0.2106
		$\hat{\gamma }$	0.9440	1.0105	0.7976	0.7909	1.2068	0.5114	1.2412	0.3158	0.6776
250	Bias	$\hat{\alpha }$	–0.0924	–0.0963	–0.0907	–0.0892	–0.1031	–0.0386	–0.1092	–0.0406	–0.0242
		$\hat{\lambda }$	–0.0866	–0.0501	–0.0646	–0.0649	–0.0600	–0.0275	–0.0802	–0.0324	–0.0108
		$\hat{\kappa }$	0.2390	0.2715	0.2328	0.2272	0.3084	0.0913	0.3356	0.1159	0.0879
		$\hat{\gamma }$	0.2813	0.1475	0.1686	0.1746	0.2603	–0.0593	0.3311	–0.0346	–0.0778
	MSE	$\hat{\alpha }$	0.0145	0.0169	0.0144	0.0137	0.0172	0.0156	0.0180	0.0187	0.0310
		$\hat{\lambda }$	0.0214	0.0240	0.0210	0.0222	0.0235	0.0239	0.0212	0.0229	0.0502
		$\hat{\kappa }$	0.1166	0.1638	0.1182	0.1133	0.1859	0.0770	0.2126	0.0925	0.0994
		$\hat{\gamma }$	0.3346	0.6233	0.3836	0.3604	0.7018	0.2243	0.5014	0.1619	0.3533
500	Bias	$\hat{\alpha }$	–0.0525	–0.0564	–0.0518	–0.0499	–0.0612	–0.0139	–0.0634	–0.0248	–0.0107
		$\hat{\lambda }$	–0.0346	–0.0077	–0.0205	–0.0179	–0.0154	0.0153	–0.0294	–0.0056	0.0251
		$\hat{\kappa }$	0.1299	0.1546	0.1301	0.1280	0.1733	0.0446	0.1933	0.0754	0.0545
		$\hat{\gamma }$	0.1196	0.0311	0.0603	0.0611	0.0887	–0.0867	0.1523	–0.0200	–0.0871
	MSE	$\hat{\alpha }$	0.0073	0.0098	0.0077	0.0074	0.0098	0.0080	0.0115	0.0104	0.0161
		$\hat{\lambda }$	0.0105	0.0155	0.0111	0.0108	0.0149	0.0152	0.0114	0.0141	0.0336
		$\hat{\kappa }$	0.0496	0.0701	0.0497	0.0479	0.0764	0.0392	0.0913	0.0476	0.0505
		$\hat{\gamma }$	0.1398	0.3087	0.1733	0.1664	0.3215	0.1288	0.2101	0.1124	0.2229
750	Bias	$\hat{\alpha }$	–0.0340	–0.0391	–0.0343	–0.0327	–0.0429	–0.0024	–0.0443	–0.0114	–0.0039
		$\hat{\lambda }$	–0.0212	–0.0033	–0.0121	–0.0097	–0.0092	0.0226	–0.0195	0.0046	0.0219
		$\hat{\kappa }$	0.0893	0.1105	0.0900	0.0883	0.1231	0.0291	0.1368	0.0523	0.0396
		$\hat{\gamma }$	0.0813	0.0223	0.0416	0.0405	0.0609	–0.0696	0.1063	–0.0196	–0.0534
	MSE	$\hat{\alpha }$	0.0055	0.0075	0.0058	0.0055	0.0075	0.0067	0.0086	0.0087	0.0096
		$\hat{\lambda }$	0.0081	0.0131	0.0091	0.0090	0.0127	0.0107	0.0095	0.0116	0.0179
		$\hat{\kappa }$	0.0308	0.0465	0.0323	0.0312	0.0495	0.0240	0.0586	0.0330	0.0313
		$\hat{\gamma }$	0.0924	0.2103	0.1156	0.1137	0.2158	0.0891	0.1363	0.0845	0.1359
1000	Bias	$\hat{\alpha }$	–0.0247	–0.0305	–0.0272	–0.0255	–0.0337	0.0022	–0.0272	–0.0061	0.0061
		$\hat{\lambda }$	–0.0126	–0.0042	–0.0099	–0.0079	–0.0090	0.0251	–0.0082	0.0097	0.0316
		$\hat{\kappa }$	0.0687	0.0883	0.0728	0.0710	0.0980	0.0204	0.0985	0.0409	0.0213
		$\hat{\gamma }$	0.0479	0.0231	0.0304	0.0287	0.0523	–0.0739	0.0648	–0.0265	–0.0759
	MSE	$\hat{\alpha }$	0.0048	0.0069	0.0052	0.0050	0.0068	0.0060	0.0085	0.0075	0.0098
		$\hat{\lambda }$	0.0065	0.0120	0.0086	0.0084	0.0117	0.0087	0.0088	0.0102	0.0181
		$\hat{\kappa }$	0.0216	0.0329	0.0243	0.0237	0.0348	0.0175	0.0430	0.0268	0.0255
		$\hat{\gamma }$	0.0616	0.1462	0.0806	0.0789	0.1493	0.0647	0.0910	0.0724	0.1134

- Based on the results presented in Table 3 and their corresponding graphical representations, it is clearly seen that as the sample size increases, the bias and MSE values decrease for all parameter estimation methods. For smaller sample sizes (n=50,100,250), the higher bias and MSE values obtained by the LSE and RTADE methods indicate that these methods produce inconsistent results under small sample sizes. On the other hand, the MPSE and MLE methods outperform the other estimation methods with lower bias and MSE measures across both small and large sample sizes. The WLSE, ADE, and CvME methods provide results comparable to these methods, whereas the LSE method demonstrates higher bias and MSE values.
- According to the results and graphical representations in Table 4, both bias and MSE values decrease as the sample size increases for all estimators. For small sample sizes (n = 50, 100, 250), the MLE and MPSE estimators generally provide the best results with the lowest bias and MSE values, while the WLSE method provides competitive estimates. In addition, the MLE and MPSE clearly outperform the other estimators in terms of both bias and MSE as the sample size increases. On the other hand, the MSALDE and RTADE methods exhibit higher bias and MSE values compared to the others and therefore tend to underperform in most sample sizes.
- When examining Table 5 and Figs 7 and 8, it is observed that the RTADE and MSALDE methods provide inconsistent results in terms of bias and MSE for small sample sizes (n=50,100,250). In contrast, the MPSE and MLE methods provide more reliable estimates with lower error values compared to other methods. As the sample size increases, the differences among the methods decrease, and particularly the MLE, MPSE, and WLSE methods demonstrate more stable and efficient performance than the other estimation methods.

## 5 A novel regression analysis

The quantile regression model is proposed by [30]. This model provides an appropriate alternative to classical regression, as it does not require any assumptions about the error terms and can be applied when the assumptions of ordinary regression are violated. This method is used to model the conditional quantiles of the response variable as a function of explanatory variables, and is particularly preferred for obtaining robust estimates in datasets containing outliers or skewed distributions. When the response variable is defined on a bounded interval, regression models based on unit distributions can be used to model its conditional mean or quantiles; among these, the beta regression model is one of the most commonly used approaches in the literature. However, when the response variable exhibits a skewed distribution, the use of the beta regression model may not be appropriate. In this case, using the median instead of the mean provides a more suitable alternative. By reparameterizing unit distributions based on the quantile function, several new regression models have been developed to model the conditional quantiles of variables bounded within the (0,1) interval. In this context, the beta [23], KW [24], UBXII [12], unit log-log [13], and log-extended exponential geometric [25] quantile regression models are proposed.In this section, a novel regression analysis based on the GMOKW distribution is introduced as an alternative to the existing regression models. The quantile function presented in Eq (7) is utilized to derive this novel regression model. Re-parameterization of the pdf and cdf of the GMOKW can be accomplished by employing the quantile function. Let Q(p;α,λ,η,δ)=μ and then

$$\kappa =\frac{\log\left(1-{\left(\frac{\lambda +p(1-\alpha )-2+\sqrt{(1-\alpha {)}^{2}p^{2}+\left((4-2\lambda )\alpha -2\lambda \right)p+\lambda^{2}}}{2(\lambda -1)}\right)}^{\frac{1}{\beta }}\right)}{\ln(\mu )}$$ (21)

is obtained and the κ in Eq (21) will be denoted as $\kappa^{*}$. The cdf and pdf of the re-parametrized distribution are derived, respectively, by

$$H\left(t;\alpha ,\lambda ,\gamma ,\mu \right)=\frac{\lambda \left(1-{\left(1-t^{\kappa^{*}}\right)}^{\gamma }\right)+\left(1-\lambda \right){\left(1-{\left(1-t^{\kappa^{*}}\right)}^{\gamma }\right)}^{2}}{\alpha +\left(1-\alpha \right)\left(1-{\left(1-t^{\kappa^{*}}\right)}^{\gamma }\right)}$$ (22)

and

$$h\left(t;\alpha ,\lambda ,\gamma \right)=\left[\frac{\left(1-\alpha \right)\left(1-\lambda \right){\left(1-{\left(1-t^{\kappa^{*}}\right)}^{\gamma }\right)}^{2}+2\alpha \left(1-\lambda \right)\left(1-{\left(1-t^{\kappa^{*}}\right)}^{\gamma }\right)+\alpha \lambda }{{\left\{\alpha +\left(1-\alpha \right)\left(1-{\left(1-t^{\kappa^{*}}\right)}^{\gamma }\right)\right\}}^{2}}\right]\;\;\times \kappa^{*}\gamma t^{\kappa^{*}-1}{\left(1-t^{\kappa^{*}}\right)}^{\gamma -1},$$ (23)

where $\kappa^{*}$ in Eq (21), 0<α,λ<1,γ>0 and μ denotes the quantile regression parameter. The *p* is chosen from (0,1) and can take values of 0.25, 0.5, or 0.75. In the remainder of the manuscript, the random variable *T* will be denoted by T~QGMOKW(α,λ,γ,μ). After defining the QGMOKW, the new regression model incorporating the pdf of the QGMOKW from Eq (23) can be introduced. Consider $T_{i}\sim QGMOKW\left(\alpha ,\lambda ,\gamma ,\mu_{i}\right)$ for i=1,2,…,n and α,λ,γ and μ are unknown parameters, and *p* is selected as 0.5. The proposed quantile regression model is formulated as follows:


$$g\left(\mu_{i}\right)={𝐱}_{i}\beta^{\text{T}}$$

where β $=(\beta_{0},\beta_{1},\ldots ,\beta_{p}{)}^{T}$ $\in {\mathbb{R}}^{p}$ are the unknown regression parameter vectors and ${𝐱}_{i}=(1,{𝐱}_{i1},{𝐱}_{i2},\ldots ,{𝐱}_{ip})$ and i=1,2,…,n known *i*th vector of the covariates and *g* is a link function. The following logit-link function is utilized since the QGMOKW is defined within the interval (0,1):

$$g\left(\mu_{i}\right)=\log\left(\frac{\mu_{i}}{1-\mu_{i}}\right),i=1,2,\ldots ,n.$$ (24)

The $\mu_{i}$ is obtained as using Eq (24)

$$\mu_{i}=\frac{\exp\left({𝐱}_{i}\beta^{\text{T}}\right)}{1+\exp\left({𝐱}_{i}\beta^{\text{T}}\right)},i=1,2,\ldots ,n.$$ (25)

### 5.1 Estimation for regression model parameters

Let $T_{1},T_{2},\ldots ,T_{n}$ be a random sample from the $QGMOKW\left(\lambda ,\kappa ,\gamma ,\mu_{i}\right)$ distribution. The corresponding log-likelihood function is expressed as

$$\ell \left(\Phi \right)=2\sum_{i=1}^{n}\log\left(\left(1-\alpha \right)\left(1-\lambda \right){\left(1-{\left(1-t_{i}^{\kappa^{*}}\right)}^{\gamma }\right)}^{2}+2\alpha \left(1-\lambda \right)\left(1-{\left(1-t_{i}^{\kappa^{*}}\right)}^{\gamma }\right)+\alpha \lambda \right)\;-2\sum_{i=1}^{n}\log\left(\alpha +\left(1-\alpha \right)\left(1-{\left(1-t_{i}^{\kappa^{*}}\right)}^{\gamma }\right)\right)+\sum_{i=1}^{n}\log\left(\kappa^{*}\gamma t_{i}^{\kappa^{*}-1}{\left(1-t_{i}^{\kappa^{*}}\right)}^{\gamma -1}\right).$$ (26)

where Φ=(α,λ,γ,β). The ML of Φ, say $\hat{\Phi }=\left(\hat{\alpha },\hat{\lambda },\hat{\gamma },{\hat{\beta }}_{0},{\hat{\beta }}_{1},\ldots ,{\hat{\beta }}_{p}\right)$, is obtained by maximizing ℓ(Φ) in Eq (26). Under certain regularity conditions, the asymptotic distribution of $\left(\hat{\Phi }-\Phi \right)$ follows a multivariate normal distribution, $N_{p+1}\left(0,J^{-1}\right)$ where *J* denotes the expected information matrix. In practice, the observed information matrix is often used instead of *J*.

The log-likelihood function in Eq (26) is maximized numerically to obtain the ML estimates of *Φ*, denoted as $\hat{\Phi }$. We employed a numerical optimization algorithm, available in the R optim function, for this purpose. The standard errors (SEs) of the parameters, reported in Table 13, are obtained from the observed information matrix. This matrix (*J*) is numerically approximated by the negative of the Hessian matrix at $\hat{\Phi }$. The variance-covariance matrix is then computed as $V=J^{-1}$, and the SEs are the square roots of the diagonal elements of this matrix.

## 6 Real-life data analyses

In this section, four real-life data analyses are carried out to demonstrate the applicability of the proposed distribution and regression model.

### 6.1 Application of new distribution without covariates

In this subsection, three real datasets are analyzed to demonstrate the applicability of the GMOKW distribution in real-world scenarios. The GMOKW is compared with several competing distributions such as Beta, KW [10], UW [11], UL [18], UBXII [12], and UR [31].

The pdfs for these competing distributions are provided in Table 6. The MLE method is used to estimate all the distribution parameters of the real datasets. The fit of the distributions is evaluated with selection criteria such as Kolmogorov-Smirnov (KS), Anderson Darling (AD), Cramer Von Mises (CVM) statistics and their corresponding p-value, as well as the Akaike Information Criterion (AIC), Bayesian Information Criterion (BIC), consistent Akaike Information Criterion (CAIC), Hannan-Quinn Information Criterion (HQIC) and −2log(ℓ) value. In terms of selection criteria, the model with the lowest KS, AD, and CvM test statistics, and the lowest −2log(ℓ), AIC, BIC, CAIC, and HQIC values is defined as the best-fitting model to datasets. On the contrary the model with the highest p-value of the KS, AD, and CvM test statistics provides the best fit to the data.

**Table 6:** The pdfs for these competing distributions.

$f_{KW}\left(x;\alpha ,\lambda \right)=\alpha \lambda x^{\alpha -1}(1-x^{\alpha }{)}^{\lambda -1}$	,	α,λ>0
$f_{UW}\left(x;\alpha ,\lambda \right)=\frac{1}{x}\alpha \lambda (-\log x{)}^{\lambda -1}\exp\left(-\alpha (-\log x{)}^{\lambda }\right)$	,	α,λ>0
$f_{UL}\left(x;\phi ,\alpha \right)=\frac{\alpha^{2}}{1+\alpha }(1-x{)}^{-3}\exp\left(-\frac{x\alpha }{1-x}\right)$	,	α>0
$f_{UBXII}\left(x;\alpha ,\lambda \right)=\frac{\alpha \lambda (-\log x{)}^{\lambda -1}(1+(-\log x{)}^{\lambda }{)}^{-\alpha -1}}{x}$	,	α,λ>0
$f_{UR}\left(x;\alpha \right)=-\frac{2\alpha }{x}\log(x)\exp\left(-\alpha \log^{2}(x)\right)$	,	α>0
$f_{Beta}\frac{1}{B\left(\alpha ,\lambda \right)}x^{\alpha -1}{\left(1-x\right)}^{\lambda -1}$	,	α>0,λ>0

**Fig 9:**
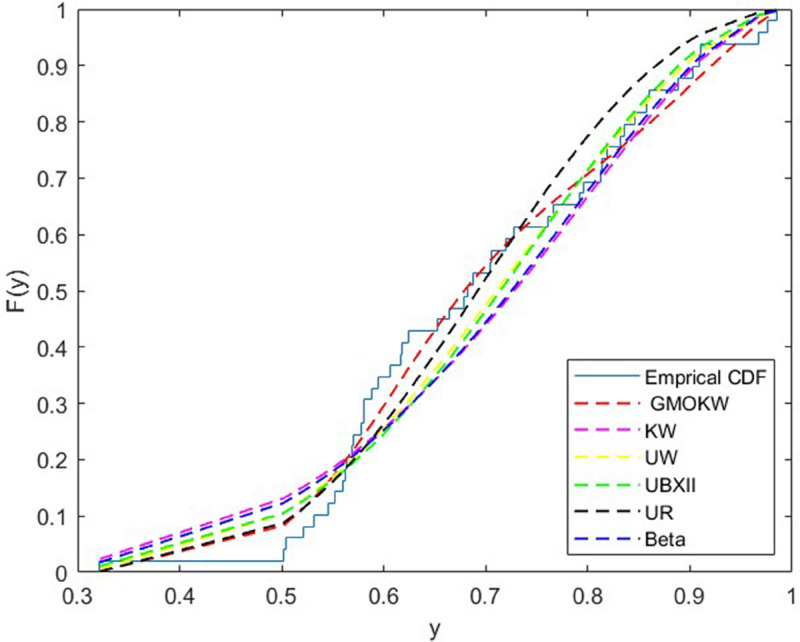
Emprical and fitted cdf plots for dataset I.

The first dataset comprises the share of Electoral College votes secured by the winning candidate in U.S. presidential elections from 1824 to 2016 [32].In Table 7, the MLEs of the fitted distributions and their corresponding SEs are reported for dataset I, while Table 8 presents the model selection criteria and relevant statistics. Moreover, the cdf plots for the fitted distributions are demonstrated in Fig 9. As seen from Table 8, the GMOKW distribution provides a better fit to the data than other distributions in terms of several criteria. This results is also supported by the comparison of the empirical and fitted cdf plots shown in Fig 6. Therefore, the GMOKW distribution can be considered as the model that best represents the empirical distribution function.

**Table 7:** The MLEs and corresponding SEs for dataset I.

Distribution	$\hat{\alpha }$	$\hat{\lambda }$	$\hat{\kappa }$	$\hat{\gamma }$	SE$\left(\hat{\alpha }\right)$	SE$\left(\hat{\lambda }\right)$	SE$\left(\hat{\kappa }\right)$	SE$\left(\hat{\gamma }\right)$
GMOKW	0.0224	0.6900	9.3896	1.9847	0.0227	0.1190	1.9461	0.7762
KW	3.9336	2.0526	–	–	0.5973	0.4238	–	–
UW	4.2207	1.6831	–	–	0.7968	0.1955	–	–
UL	0.3144	–	–	–	0.0322	–	–	–
UBXII	5.5219	1.8477	–	–	1.0204	0.2024	–	–
UR	5.1101	–	–	–	0.7300	–	–	–
Beta	4.8950	2.0533	–	–	0.9916	–	–	0.3879

**Table 8:** The modeling results for all distributions on dataset I.

Distribution	–2 log (ℓ)	AIC	BIC	CAIC	HQIC	KS	AD	CVM	p(KS)	p(AD)	p(CvM)
GMOKW	**—54.5747**	**—46.5747**	–39.0074	**—45.6656**	–43.7037	**0.0669**	**0.4077**	**0.0485**	**0.9703**	**0.8404**	**0.8876**
KW	–44.5502	–40.5502	–36.7666	–40.2893	–39.1147	0.1340	1.2653	0.2097	0.3138	0.2438	0.2498
UW	–49.5728	–45.5728	**–41.7891**	–45.3119	**–44.1372**	0.1249	0.8633	0.1344	0.3965	0.4369	0.4423
UL	12.1131	14.1131	16.0049	14.1982	14.8308	0.3194	9.4476	1.7550	0.0001	0.0000	0.0000
UBXII	–47.5409	–43.5409	–39.7573	–43.2800	–42.1054	0.1363	1.0617	0.1657	0.2948	0.3261	0.3459
UR	–47.1707	–45.1707	–43.2788	–45.0856	–44.4529	0.1084	1.2593	0.1502	0.5751	0.2459	0.3901
Beta	–45.6753	–41.6753	–37.8917	–41.4145	–40.2398	0.1355	1.1865	0.1953	0.3013	0.2725	0.2772

**Fig 10:**
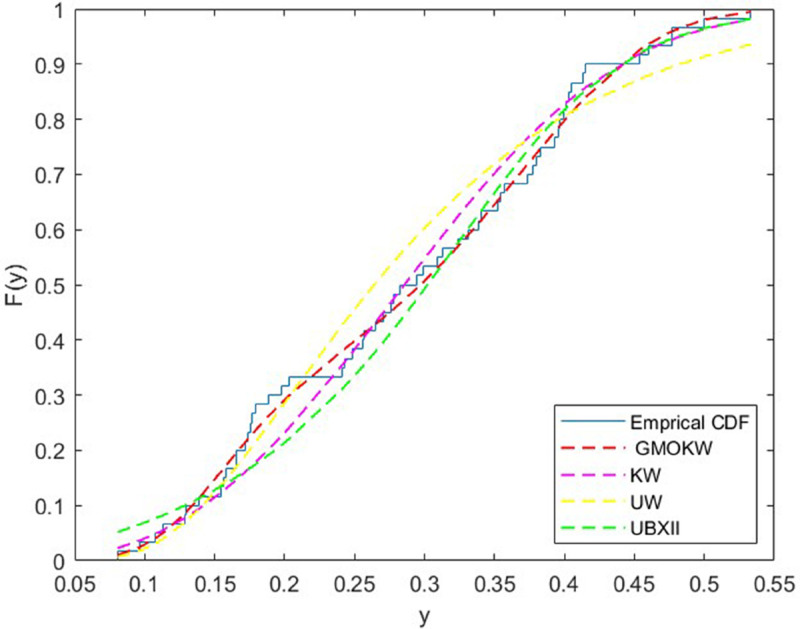
Emprical and fitted cdf plots for dataset II.

The second dataset comprises the mortality rates in the United Kingdom over a 60-day period [33]. In Table 9, the MLEs of the fitted distributions and their corresponding SEs are reported for dataset II, while Table 10 presents the model selection criteria and relevant statistics. Moreover, the cdf plots for the fitted distributions are demonstrated in Fig 10. According to Table 10, the GMOKW model provides the best fit to the data for all selection criteria except the BIC when compared with the competing models. This finding, supported by the empirical and fitted CDF plots presented in Fig 10, indicates that the GMOKW distribution best represents the empirical distribution function.

**Table 9:** The MLEs and corresponding SEs for dataset II.

Distribution	$\hat{\alpha }$	$\hat{\lambda }$	$\hat{\kappa }$	$\hat{\gamma }$	SE$\left(\hat{\alpha }\right)$	SE$\left(\hat{\lambda }\right)$	SE$\left(\hat{\kappa }\right)$	SE$\left(\hat{\gamma }\right)$
GMOKW	0.0145	0.3825	4.9552	106.5613	0.0124	0.0873	0.7126	64.8097
KW	2.6806	19.5873	–	–	0.3009	6.5008	–	–
UW	0.2834	3.1229	–	–	0.0602	0.3047	–	–
UL	2.8294	–	–	–	0.3029	–	–	–
UBXII	0.4637	6.9086	–	–	0.0856	1.1029	–	–
UR	0.5000	–	–	–	0.0646	–	–	–
Beta	1.4096	1.3895	–	–	0.1555	0.1530	–	–

**Table 10:** The modeling results for all distributions on dataset II.

Distribution	–2 log (ℓ)	AIC	BIC	CAIC	HQIC	KS	AD	CvM	p(KS)	p(AD)	p(CvM)
GMOKW	**–99.1629**	**–91.1629**	–82.7855	**–90.4357**	**–87.8861**	**0.0594**	**0.1777**	**0.0254**	**0.9754**	**0.9954**	**0.9893**
KW	–91.7288	–87.7288	**–83.5401**	–87.5183	–86.0904	0.1054	0.6298	0.1047	0.4847	0.6196	0.5639
UW	–85.1244	–81.1244	–76.9357	–80.9138	–79.4859	0.0955	1.0653	0.1819	0.6105	0.3245	0.3059
UL	–64.7546	–62.7546	–60.6602	–62.6856	–61.9354	0.2061	4.4878	0.7574	0.0103	0.0051	0.0090
UBXII	–86.4749	–82.4749	–78.2862	–82.2644	–80.8365	0.1134	0.7381	0.1118	0.3940	0.5272	0.5313
UR	–68.4261	–66.4261	–64.3318	–66.3572	–65.6069	0.2202	3.4745	0.5552	0.0049	0.0159	0.0286
Beta	–9.9722	–5.9722	–0.0187	–5.8877	–3.5531	0.11178	2.6440	0.4667	0.0357	0.0417	0.0483

**Fig 11:**
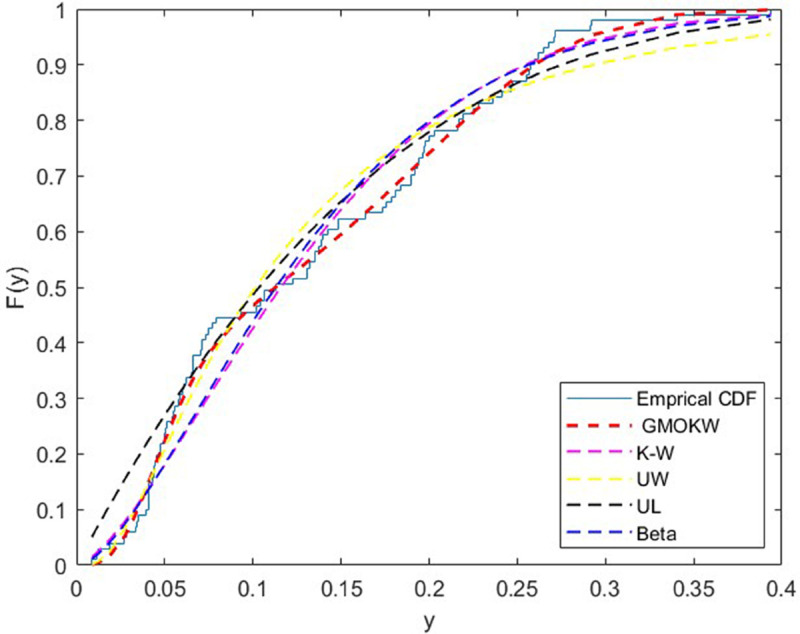
Emprical and fitted cdf plots for dataset III.

The third dataset belongs to the Foulum region of Denmark and is acquired through radar imagery collected by the EMISAR sensor. This dataset contains 101 observations; each data point corresponds to the squared modulus of complex-valued radar returns, which represent backscatter intensity under horizontal polarization. The dataset was previously studied by authors [34] and [35]. In Table 11, the MLEs of the fitted distributions and their corresponding SEs are reported for dataset II, while Table 12 presents the model selection criteria and relevant statistics. Moreover, the cdf plots for the fitted distributions are demonstrated in Fig 11. As shown in Table 12, the GMOKW model yields the lowest information criterion values and the lowest test statistics compared with the competing models.

**Table 11:** The MLEs and corresponding SEs for dataset III.

Distribution	$\hat{\alpha }$	$\hat{\lambda }$	$\hat{\kappa }$	$\hat{\gamma }$	SE$\left(\hat{\alpha }\right)$	SE$\left(\hat{\lambda }\right)$	SE$\left(\hat{\kappa }\right)$	SE$\left(\hat{\gamma }\right)$
GMOKW	0.0100	0.4869	3.2057	123.6860	0.0061	0.0616	0.3294	57.2755
KW	1.4635	15.8755	–	–	0.1275	3.8435	–	–
UW	0.0567	3.0240	–	–	0.0153	0.2271	–	–
UL	6.8283	–	–	–	0.6134	–	–	–
UBXII	0.0731	17.7018	–	–	0.0380	9.0417	–	–
UR	0.1673	–	–	–	0.0167	–	–	–
Beta	1.7331	11.4277	–	–	0.2257	1.6821	–	–

**Table 12:** The modeling results for all distributions on dataset III.

Distribution	–2 log (ℓ)	AIC	BIC	CAIC	HQIC	KS	AD	CvM	p(KS)	p(AD)	p(CvM)
GMOKW	**–245.8094**	**–237.8094**	**–227.3887**	**–237.3884**	**–233.5920**	**0.0478**	**0.3260**	**0.0324**	**0.9763**	**0.9172**	**0.9684**
KW	–228.1241	–224.1241	–218.9137	–224.0004	–222.0154	0.1174	1.5209	0.2739	0.1270	0.1716	0.1604
UW	–220.5779	–216.5779	–211.3676	–216.4542	–214.4692	0.1044	1.5293	0.2354	0.2254	0.1697	0.2083
UL	–219.6047	–217.6047	–214.9996	–217.5639	–216.5504	0.1274	2.2094	0.3019	0.0777	0.0708	0.1335
UBXII	–154.3474	–150.3474	–145.1371	–150.2237	–148.2387	0.2842	15.0748	2.9985	0.0000	0.0000	0.0000
UR	–196.2111	–194.2111	–191.6059	–194.1702	–193.1567	0.2074	5.2469	0.8077	0.0004	0.0022	0.0069
Beta	–228.1283	–224.1283	–218.9180	–224.0046	–222.0196	0.1088	1.5177	0.2739	0.1871	0.1723	0.1604

### 6.2 Application of new regression model with covariates

In this subsection, a real data analysis is performed to evaluate the usability and superiority of the new regression model. The dataset consists of the percentage of educational attainment in OECD countries, along with components such as voter turnout percentage, homicide rate, and life satisfaction. The data is accessible at https://stats.oecd.org/, and more detailed information can be found in [22].

For comparison purposes, the beta regression (BR), Kumaraswamy regression (KR), and log-extended exponential geometric regression (LEEG) models [25] are considered. This application aims to examine the relationship between the percentage of education level values of OECD countries (variable *y*) and voter participation percentage (variable *x*_1_), homicide rate (variable *x*_2_), and life satisfaction (variable *x*_3_).

For all models, ML, SE, $\hat{\ell }$, and AIC are calculated. The results are presented in Table 13. Table 13 shows that the QGMOKW model has the best modeling capacity and can be considered an alternative to the beta and Kumaraswamy regression models in the literature.

**Table 13:** Data analysis results for regression real data.

Parameters	QGMOKW			BR			KR			LEEG		
	Estimate	SE	p-value	Estimate	SE	p-value	Estimate	SE	p-value	Estimate	SE	p-value
$\beta_{0}$	0.9455	2.0697	0.6761	0.9615	0.9685	0.3208	1.6318	1.1629	0.1606	0.3269	1.0711	0.7602
$\beta_{1}$	–2.5397	1.5588	0.0516	–2.9211	1.0176	0.0041	–4.1024	1.3947	0.0033	–4.0914	1.4627	0.0052
$\beta_{2}$	–0.0609	0.0280	0.0147	–0.0470	0.0178	0.0083	–0.0405	0.0167	0.0153	–0.0476	0.0145	0.0010
$\beta_{3}$	0.3695	0.2018	0.9664	0.3794	0.1492	0.0110	0.4206	0.2532	0.0967	0.6215	0.1746	<0.0001
*α*	0.9853	4.3351	0.5898	11.5900	2.6100	<0.0001	6.2166	1.0844	<0.0001	7.8374	1.7424	<0.0001
*λ*	0.5787	1.3987	0.6604									
*γ*	3.3220	1.4471	0.9891									
$\hat{\ell }$	33.3687			30.9024			29.4339			28.6481		
AIC	–52.7374			–51.8048			–48.8678			–47.2962		

For the QGMOKW model, the intercept $\beta_{0}$ is estimated at 0.9455 with a p-value of 0.6761, which is not statistically significant at the 0.05 level.When all predictors are zero, the expected response is approximately 0.9455, but this estimate is not reliable due to its insignificance. The coefficient $\beta_{1}$ is estimated at -2.5397 with a p-value of 0.0516, which is very close to the 0.05 significance level but does not strictly meet the threshold. This suggests a possible negative relationship between the corresponding predictor and the response variable; however, the statistical evidence remains weak. In contrast, $\beta_{2}$ is estimated at -0.0609 with a p-value of 0.0147, indicating statistical significance at the 0.05 level. This suggests that a one-unit increase in the predictor corresponding to $\beta_{2}$ is associated with an average decrease of 0.0609 units in the response variable. The coefficient $\beta_{3}$ is estimated at 0.3695 with a p-value of 0.9664, making it highly insignificant. This implies that the corresponding predictor does not have a meaningful effect on the response variable.

Furthermore, the parameter *α* is estimated at 0.9853 with a p-value of 0.5898, indicating that it is not statistically significant. Similarly, *λ* is estimated at 0.5787 with a p-value of 0.6604, showing no significant effect. The parameter *γ* is estimated at 3.3220 with a p-value of 0.9891, making it highly insignificant. Thus, it does not contribute meaningfully to the model.

**Table 14:** The first dataset: Electoral College vote shares in U.S. presidential elections (1824-2016).

0.3218	0.6820	0.7657	0.5782	0.7959	0.6182	0.5621	0.8581	0.5878	0.5941	0.9099	0.7279
0.8125	0.5014	0.5799	0.5461	0.5810	0.6239	0.6063	0.6523	0.7059	0.6646	0.8192	0.5217
0.7608	0.7194	0.8362	0.8889	0.9849	0.8456	0.8136	0.5706	0.8324	0.8606	0.5642	0.9033
0.5595	0.9665	0.5520	0.9089	0.9758	0.7918	0.6877	0.7045	0.5037	0.5316	0.6784	0.6171
0.5687											

Overall, the only statistically significant parameter at the 0.05 level is $\beta_{2}$, which suggests that its corresponding predictor has a meaningful negative effect on the response variable. Other parameters, including $\beta_{0}$, $\beta_{1}$, $\beta_{3}$, *α*, *λ*, and *γ*, do not show strong evidence of influence in the model.

## 7 Conclusion

In this study, a new unit distribution referred to as the GMOKW distribution is introduced. Some mathematical properties of the proposed distribution, such as moments, quantile function, and Lorenz and Bonferroni curves, are investigated. Nine different estimators are considered to obtain the unknown parameters of the model, and the performance of these estimators is evaluated through a Monte Carlo simulation study. According to the Monte Carlo simulation results, in both small and large sample sizes, the MLE and MPSE methods outperform other estimator methods. Moreover, a GMOKW-based quantile regression model is proposed to model the conditional quantiles of the response variable as a function of explanatory variables. To evaluate the applicability of the proposed distribution and regression model, four real data analyses are conducted. Three datasets are utilized to evaluate the performance of the proposed distribution, whereas one dataset is used to implement the GMOKW-based quantile regression model. The results obtained from real data analyses indicate that the GMOKW distribution can be used as an alternative to other bounded distributions in modeling datasets. In addition, the GMOKW-based quantile regression model exhibits high flexibility and efficiency in modeling the association between bounded response variables and covariates.

In the light of the obtained results, it is evaluated that the proposed distribution is applicable in modeling bounded data sets obtained from various fields such as medicine, education, engineering, physics, economics and actuarial science. The GMOKW model provides a more accurate and flexible for representing bounded data such as proportions, success probabilities, and risk levels. In terms of fitting performance and interpretability, the new distribution and its corresponding regression model can be used as a powerful and flexible method for analyzing real-world bounded datasets across various disciplines, providing a strong alternative to well-known unit distributions such as the KW, Beta, UW, and UBXII distributions. Future studies may focus on extending the proposed model to different censoring schemes and developing more efficient estimation techniques.

## Appendix

The datasets used for the real-life data analysis in Sect 6 are presented below.

**Table 15:** The second dataset: Mortality rates in the United Kingdom over a 60-day period.

0.1292	0.3805	0.4049	0.2564	0.3091	0.2413	0.1390	0.1127	0.3547	0.3126	0.2991	0.2428
0.2942	0.0807	0.1285	0.2775	0.3311	0.2825	0.2559	0.2756	0.1652	0.1072	0.3383	0.3575
0.2708	0.2649	0.0961	0.1565	0.1580	0.1981	0.4154	0.3990	0.2483	0.1762	0.1760	0.1543
0.3238	0.3771	0.4132	0.4602	0.3523	0.1882	0.1742	0.4033	0.4999	0.3930	0.3963	0.3960
0.2029	0.1791	0.4768	0.5331	0.3739	0.4015	0.3828	0.1718	0.1657	0.4542	0.4772	0.3402

**Table 16:** The third dataset: Radar imagery collected by the EMISAR sensor in the Foulum region.

0.1808583885431290	0.2715894281864170	0.1936905533075330	0.0500552132725716	0.1485721915960310
0.2600140273571010	0.1304957568645480	0.0394391231238842	0.1323543041944500	0.1426382064819340
0.1129530519247060	0.0269157197326422	0.1067145913839340	0.1392973661422730	0.1899524927139280
0.0408989638090134	0.0712180733680725	0.2190646529197690	0.2168500870466230	0.0581964328885078
0.0793552622199059	0.2000004947185520	0.1894772350788120	0.0580895692110062	0.1066346019506450
0.1789414286613460	0.3403762280941010	0.0606064833700657	0.1967413872480390	0.1636120975017550
0.3936370909214020	0.0446394421160221	0.2471709847450260	0.0708507150411606	0.2923302650451660
0.0352656245231628	0.1918695569038390	0.0410193651914597	0.2552164196968080	0.0405378639698029
0.2030045837163930	0.0735185071825981	0.2565241158008580	0.0408119037747383	0.1835628747940060
0.1043748185038570	0.2619580626487730	0.0332400277256966	0.1022637560963630	0.1391180157661440
0.1960394680500030	0.0511302277445793	0.0658624023199081	0.1735171675682070	0.1375353783369060
0.0748695135116577	0.0659949705004692	0.2414010912179950	0.0767071023583412	0.0701084062457085
0.0655298158526421	0.2916960418224330	0.0188371986150742	0.0451735183596611	0.0934262275695801
0.2644947469234470	0.0086520370095968	0.0497028753161430	0.1310033947229390	0.2680946886539460
0.0113833881914616	0.0558364242315292	0.1225134804844860	0.2704213559627530	0.0123042017221451
0.0474526733160019	0.1350584030151370	0.2464897632598880	0.0281006377190351	0.0475621223449707
0.1977708339691160	0.1754100024700160	0.0552334710955620	0.0600663721561432	0.2574170231819150
0.1352893263101580	0.0505803674459457	0.0659491419792175	0.2282601296901700	0.1485791504383090
0.0341098383069038	0.0429943427443504	0.2165334820747380	0.1938208043575290	0.0539951920509338
0.0441263243556023	0.2357383966445920	0.2253356277942660	0.0620105266571045	0.0437876060605049
0.0405787900090218				
